# The Broad Aryl Acid Specificity of the Amide Bond Synthetase McbA Suggests Potential for the Biocatalytic Synthesis of Amides

**DOI:** 10.1002/anie.201804592

**Published:** 2018-08-07

**Authors:** Mark Petchey, Anibal Cuetos, Benjamin Rowlinson, Stephanie Dannevald, Amina Frese, Peter W. Sutton, Sarah Lovelock, Richard C. Lloyd, Ian J. S. Fairlamb, Gideon Grogan

**Affiliations:** ^1^ Department of Chemistry University of York York YO10 5DD UK; ^2^ GSK Medicines Research Centre Gunnels Wood Road Stevenage Hertfordshire SG1 2NY UK; ^3^ Current address: Department of Chemical, Biological and Environmental Engineering Bioprocess Engineering and Applied Biocatalysis Group Engineering School Campus de la UAB 08193 Bellaterra (Cerdanyola del Vallés) Barcelona Spain; ^4^ Current address: School of Chemistry University of Manchester Manchester Institute of Biotechnology 131 Princess Street Manchester M1 7DN UK

**Keywords:** adenylation, amides, ATP, biocatalysis, ligases

## Abstract

Amide bond formation is one of the most important reactions in pharmaceutical synthetic chemistry. The development of sustainable methods for amide bond formation, including those that are catalyzed by enzymes, is therefore of significant interest. The ATP‐dependent amide bond synthetase (ABS) enzyme McbA, from *Marinactinospora thermotolerans*, catalyzes the formation of amides as part of the biosynthetic pathway towards the marinacarboline secondary metabolites. The reaction proceeds via an adenylate intermediate, with both adenylation and amidation steps catalyzed within one active site. In this study, McbA was applied to the synthesis of pharmaceutical‐type amides from a range of aryl carboxylic acids with partner amines provided at 1–5 molar equivalents. The structure of McbA revealed the structural determinants of aryl acid substrate tolerance and differences in conformation associated with the two half reactions catalyzed. The catalytic performance of McbA, coupled with the structure, suggest that this and other ABS enzymes may be engineered for applications in the sustainable synthesis of pharmaceutically relevant (chiral) amides.

The formation of amide bonds is one of the most important reactions in pharmaceutical synthetic chemistry, accounting for up to 16 % of all synthetic steps in medicinal chemistry laboratories.^1^ Amide bond synthesis typically involves activation of a carboxylic acid, using either toxic chlorinating agents, or atom‐inefficient coupling reagents,^2^ and so there is an urgent need to investigate methods for amide bond formation that are sustainable in terms of reagent safety and atom efficiency. Enzymatic methods for amide bond synthesis that display these advantages are thus appealing.^3^, ^4^ Amide bonds are encountered in secondary metabolites that are synthesized by non‐ribosomal peptide synthases (NRPSs).^5^ In this case, the formation of the amide is complex, requiring ATP‐dependent formation of an intermediate adenylate of the carboxylic acid substrate, followed by transfer to a phosphopantetheine thiol enabled by an acyl/peptidyl carrier protein (ACP or PCP), followed by condensation with the amine substrate, with each process taking place in a separate domain of a large, multidomain NRPS enzyme. While NRPS‐mediated amide bond formation may have a role in synthetic biology pathways towards amides,^6^ their structural and catalytic complexity militates against their application in preparative in vitro biocatalysis. Hydrolases, such as N‐acylases^7^ and lipases,^8^ have been employed for amide bond formation and are attractive in terms of their simplicity and efficiency, but they often require ester substrates and suffer from poor substrate scope. More recently, Flitsch and co‐workers have recruited the adenylate‐forming ability of a carboxylate reductase (CAR) adenylation‐domain‐plus‐peptidyl carrier protein (CAR‐A‐PCP) to the formation of amides, although a 100‐fold excess of amine was required to drive reactions to high conversions.^9^

ATP‐dependent amide bond synthetases (ABSs) have been discovered in secondary metabolite pathways leading to compounds such as novobiocin,^10^ cloromycin,^11^ and coumermycin.^12^ The enzymes NovL and CouL, for example, couple the aminoflavone **1** with carboxylic acids **2** and **4** in their respective biosynthetic pathways to give amide products **3** and **5** (Scheme 1 A).

**Scheme 1:**
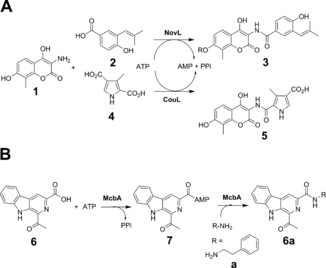
A) Amide bond synthesis by ATP‐dependent amide bond synthetases (ABSs) NovL and CouL involved in secondary metabolism in actinomycetes. B) Formation of marinacarbolines such as **6 a** from β‐carboline acid **6** and amine **a** catalyzed by McbA from *Marinactinospora thermotolerans*.

These enzymes are apparently capable of catalyzing formation of the adenylate, but also of binding the amine for the coupling reaction within the same active site, with no involvement of separate acyl carrier protein or condensation domains. A further enzyme, McbA, has recently been reported in the biosynthetic pathway towards the marinacarbolines in *Marinactinospora thermotolerans* by Ji and co‐workers.^13^, ^14^ McbA catalyzes the ATP‐dependent coupling of the β‐carboline derivative **6** with 2‐phenylethylamine **a** to form **6 a** via adenylate **7** (Scheme 1 B), but, unlike other ABSs, McbA also accepted other amines, including substituted 2‐phenylethylamines and tryptamines, thus indicating some promise for the application of this enzyme to reactions with a wider substrate spectrum.^14^ These studies prompted us to further examine the activity of McbA to include a range of β‐carboline and other aryl carboxylic acid acceptors. We also determined the structure of McbA in complex with AMP and **6**, which revealed the substrate binding interactions within the active site, and may serve as a platform for protein engineering to expand the substrate specificity of this and related ABS enzymes.

McbA was expressed from a synthetic gene and the protein purified using IMAC followed by gel filtration (Figures S1, S2 in the Supporting Information). A sample of the pure enzyme (1 mg mL^−1^; 9.4 nmol) was applied to the coupling of 0.4 mm acid **6** and 0.6 mm 2‐phenylethylamine **a**, in the presence of 2 mm ATP on an analytical scale, and full conversion to product amide **6 a** was observed within 1 h using HPLC. The standard amide product was prepared by reacting the β‐carboline ester with the amine in DMSO at 90 °C (see the Supporting Information). We then further explored the constraints on substrate structure in the McbA‐catalyzed reaction, but, in contrast to previous work,^14^ we focused on the structure of the carboxylic acid acceptor (Scheme 2).

**Scheme 2:**
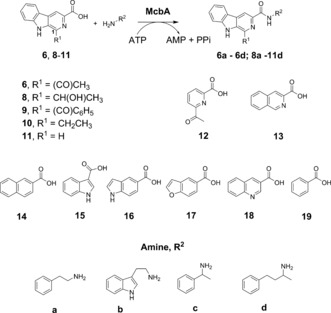
β‐carboline carboxylic acid and amine partners for McbA‐catalyzed amide bond formations.

Carboxylic acid substrates **6** and **8**–**11** were synthesized using Pictet–Spengler condensations of tryptophan‐derived esters with relevant aldehydes, followed by hydrolysis of the ester products, as detailed in the Supporting Information. Substitution of the acyl group in position 1 of the pyridine ring with a secondary hydroxymethyl (**8**) reduced the conversion to 21 % of product **8 a** after 1 h, but 96 % conversion was achieved after 24 h. An increase in the size of the substituent to benzoyl **9** gave conversions to product **9 a** of 8 % and 82 % after 1 h and 24 h, respectively. Replacement by an ethyl group **10** showed that an oxygen atom on this substituent was not necessary, with 100 % conversion to product **10 a** again achieved after 24 h. Even replacement with H (**11**) resulted in 6 % conversion to **11 a** after 24 h. The activity of the enzyme was then tested using carboxylic acids **6** and **8**–**11** with amines **a**–**d** (Scheme 2, Table 1).

**Table 1:** ATP‐dependent amide couplings by McbA.^[a]^

	Amine			
Acid	**a**	**b**	**c**	**d**
**6**	100	81	6	26
**8**	96	100	0	17
**9**	82	0	0	0
**10**	100	91	0	0
**11**	6	0	0	0

[a] Values represent % conversions to amide products after 24 h as determined by HPLC analysis.

McbA catalyzed the formation of **6 a** and **6 b** with 100 % and 81 % conversion, respectively, thus confirming the findings of Ji and co‐workers.^14^ However, McbA also catalyzed the coupling of **6** to the racemic chiral amines **c** and **d** to form **6 c** and **6 d**, although with lower conversions of 6 % and 26 %. Interestingly, analysis of the product **6 d** by chiral HPLC revealed it to be predominantly of (*S*)‐configuration, and with an *ee* of 96 % (Figure S3), thus indicating that McbA is capable of performing kinetic resolution reactions. Tryptamine (**b**) was also coupled to **10** to give **10 b** in 91 % yield. 1‐Phenylethylamine (**c**) proved to be a poor donor overall, but 3‐phenyl‐1‐methylpropylamine **d** was coupled with **8** to form amide **8 d** with a conversion of 17 %. Some of the β‐carboline amides synthesized here have been shown to have biological activity, most notably against benzodiazepine receptors, which was first reported in the 1980s,^15^ and are of interest as treatments for alcohol abuse, for example.^16^

In order to investigate the broader applicability of McbA to amide bond synthesis, aryl acid substrates **12**–**19**, which have structures more diverged from the β‐carboline acid platform, were then tested with 2‐phenylethylamine (**a**) in an effort to produce amide products **12 a** to **19 a** (Table 2).

**Table 2:** ATP‐dependent coupling of **12**–**19** with amine **a** by McbA.^[a]^

Acid	Product	Conversion [%]
12	**12 a**	34
13	**13 a**	23
14	**14 a**	43
15	**15 a**	40
16	**16 a**	41
17	**17 a**	39
18	**18 a**	4
19	**19 a**	24

[a] Values represent % conversions to amide products after 24 h as determined by HPLC analysis.

Overall, McbA displayed a surprisingly broad substrate range, including substrates such as naphthoic acid **14**, indole carboxylic acids **15** and **16**, and benzofuran‐5‐carboxylic acid **17**, although quinoline substrate **18** was transformed with low conversion. Strikingly, McbA was also able to couple benzoic acid **19** with **a** with a modest but significant conversion of 24 %.

To investigate the promiscuity in term of aryl acid recognition by McbA, the structure of the enzyme was determined using X‐ray crystallography. A K483A mutant co‐crystallised with AMP, magnesium ions, and the β‐carboline acid substrate **6** yielded crystals of diffraction quality. The structure was solved using molecular replacement, and refined to a resolution of 2.80 Å (data collection and refinement statistics can be found in Table S2 in the Supporting Information). McbA adopts a structure within the ANL superfamily of adenylating enzymes defined by Gulick,^17^ and which includes firefly luciferase,^18^ acetyl‐CoA synthase,^19^ and standalone adenylation domains of NRPSs, including the dihydroxybenzoate adenylating enzyme enzyme DhbE^20^ from the bacillibactin biosynthetic pathway. Each ANL enzyme is characterized by a two‐domain structure in which a large N‐terminal domain is coupled to a smaller C‐terminal one, consisting of 4–500 and 100 amino acid residues, respectively. Crucially, the structures of many ANL enzymes have revealed substantial relative domain rotation during the reaction coordinate, with one conformation (“adenylation”) assumed for adenylate formation from the carboxylate substrate and ATP, and a second, (“thiolation”) in which the C‐terminal domain rotates to enable the ligation reaction between the phosphopantetheinate thiol and the adenylate intermediate.

The McbA structure features the two predicted domains: the large N‐terminal domain of residues 1–394 (McbA_N_) and the smaller C‐terminal one comprising residues 395–494 (McbA_C_), with the hinge residue at Q394. The McbA structure is unusual however, in that, of five molecules within the asymmetric unit, four (A–D) are in the “adenylation” conformation (Figure 1 a) and one (E) is in the “thiolation” state described above, which we have termed “amidation” (Figure 1 b) for McbA. The adenylation conformer of McbA superimposes well with other ANL enzymes crystallized in the same state, including PheA (PDB ID: 1AMU, 2.1 Å over 431 Cα atoms),^21^ DhbE (PDB ID: 1MD9, 2.3 Å over 445),^20^ and the recently determined structure of the relevant “A” domain from CAR from *Nocardia iowenis* (*Ni*CAR) in complex with benzoic acid and AMP (PDB ID: 5MSD, 2.8 Å over 406).^22^

**Figure 1:**
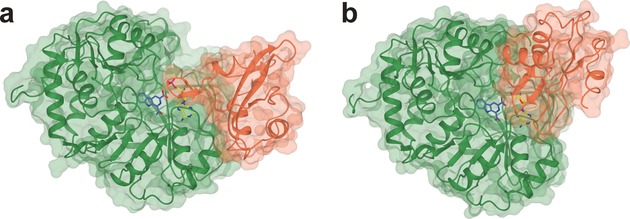
Structures of McbA in the adenylation (a) and amidation (b) conformations. In each case the protein is colored in green from residues 1–394 (McbA_N_) and coral from 395–494 (McbA_C_). AMP (yellow) is bound at the domain interface and **6** (blue) within a binding pocket in the N‐terminal domain. The McbA_C_ domain in (b) is rotated 149° relative to the larger McbA_N_ domain compared with their orientations in (a).

The substrate **6** is bound within a hydrophobic pocket stacked between the loop between G295 and F301 on one face and L202 on the other (Figure 2). Few specific interactions between active‐site residues and the substrate are made, except that the carbonyl oxygen and pyridine nitrogen of **6** interact with the backbone N‐H and C=O of G295 at distances of approximately 3.9 and 3.4 Å respectively. The relative lack of specific interactions may help to explain the relaxed specificity of McbA for the range of aryl acids used in this study. The exception was quinoline derivative **18**, in which the N‐atom in the 1‐position has had an adverse effect on conversion, perhaps disrupting the hydrophobic interaction made by the native ligand with F301. Residue D201, which points away from **6** in the active site, is replaced in CARs and DhbE by H300, and is close to the benzoic acid carboxylate in the CAR–AMP complex 5MSD. This histidine has been implicated in the catalysis of both adenylation and thiolation reactions in 4‐chlorobenzoyl‐CoA ligase (PDB IDs: 4CBL, 3CW8, 3CW9).^23^, ^24^

**Figure 2:**
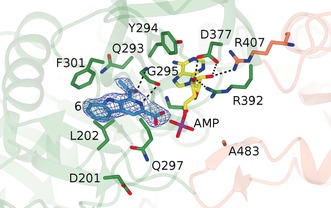
Active site of the adenylation conformer of McbA showing residues involved in the binding of substrate **6** and AMP. Electron density is the *F*_o_−*F*_c_ (omit) map at a level of 3*σ* before inclusion of the ligand atoms in refinement. Ligand atoms have been added for clarity. Selected interactions are indicated by black dashed lines.

The amidation conformer of McbA superimposes well with the thiolation conformer of 4‐CBL (PDB ID: 3CW9, 2.0 Å over 430 Cα atoms) and also the “A” domain in the structure of CAR‐A‐PCP (PDB ID: 5MSS, 2.5 Å over 402), the construct used in amide bond forming reactions described previously.^9^ The structure of 4‐CBL in the thiolation state, in complex with 4‐chlorophenacyl‐ CoA (4‐CPA‐CoA, PDB ID: 3CW9; Figure S3a)^23^ identifies the binding site for the thiolation product, and permits a comparison with the equivalent region of McbA (Figure S3b) The surfaces of the enzymes show that positively charged residues in 4‐CBL, such as R87, which interact with the phosphates in 4‐CPA‐CoA, are absent in McbA; indeed the entrance to the active site features three residues with carboxylate side chains (D463, E221 and E400), which may favor interaction with incoming amine substrates.

The McbA‐catalyzed synthesis of selected amides was scaled up to utilize 50 mg (0.20 mmol) of aryl acids **6**, **10**, **14**, **16**, **17**, and **19** in a 50 mL reaction volume, with 1–5 molar equivalents 2‐phenylethylamine (**a**) and two equivalents of ATP (198 mg; 0.40 mmol). Amide products **6 a**, **10 a**, **14 a, 17 a**, and **19 a** were isolated by simple liquid‐liquid extraction into ethyl acetate, giving isolated yields of between 50 % and 85 % of the amide products without further purification (Table 3). **16 a** was also isolated, although only in 15 % yield owing to complications in extraction of this indole product. These results compare favorably with the application of CAR‐A‐PCP to preparative amide bond formation,^9^ since 100 equivalents of amine donor were required for those reactions, compared to the low ratios reported here. The difference may reflect of the active participation of McbA in amine binding and catalysis of amidation, which was not yet established for the CAR‐A‐PCP reaction. The results offer promise for the application of McbA‐type enzymes in preparative biocatalyzed reactions, particularly if ATP recycling methods, currently a subject of significant interest in biocatalysis,^25^ were to be employed.

**Table 3:** ATP‐dependent coupling of carboxylic acids with amine **a** by McbA on 50 mg scale.

Acid	Equivalents of amine	Yield of isolated product [%]
**6**	2	85 (**6 a**)
**10**	2	70 (**10 a**)^**[a]**^
**14**	2	50 (**14 a**)
**16**	5	15 (**16 a**)
**17**	5	51 (**17 a**)
**19**	5	21 (**19 a**)

[a] carried out on 30 mg scale

The efficient biocatalytic formation of amide bonds for the preparation of pharmaceutical‐type amides is one of the most significant reactions for which biocatalytic alternatives are being sought. The studies presented here suggest that McbA and related ABS enzymes may provide starting points for the evolution of activity towards a wider range of carboxylic acid and amine partners, using the structure of McbA as a basis for structure‐guided evolution.

## Experimental Section

Details of target selection, gene cloning and expression, enzyme purification and assay, synthesis of substrates and product standards, HPLC analyses, biotransformation protocols, crystallisation and data collection and refinement can be found in the Supporting Information.

## Conflict of interest

The authors declare no conflict of interest.

## Supporting information

As a service to our authors and readers, this journal provides supporting information supplied by the authors. Such materials are peer reviewed and may be re‐organized for online delivery, but are not copy‐edited or typeset. Technical support issues arising from supporting information (other than missing files) should be addressed to the authors.

Supplementary
