# Supplementary material for: The Broad Aryl Acid Specificity of the Amide Bond Synthetase McbA Suggests Potential for the Biocatalytic Synthesis of Amides
Source: Angew Chem Int Ed Engl. 2018 Aug 7;57(36):11584–8. doi: 10.1002/anie.201804592 (PMC6282839; doi:10.1002/anie.201804592)
Supplement: Supplementary file 1 — Supplementary [file ANIE-57-11584-s001.pdf]

Angewandte Chemie
Eine Zeitschrift der Gesellschaft Deutscher Chemiker

Supporting Information

The Broad Aryl Acid Specificity of the Amide Bond Synthetase McbA Suggests Potential for the Biocatalytic Synthesis of Amides

Mark Petchey, Anibal Cuetos, Benjamin Rowlinson, Stephanie Dannevald, Amina Frese, Peter W. Sutton, Sarah Lovelock, Richard C. Lloyd, Ian J. S. Fairlamb, and Gideon Grogan**

anie_201804592_sm_miscellaneous_information.pdf

Table of Contents

1. General information..3

2. Gene cloning, expression and protein purification..4

3. Synthesis of Substrates and Product Standards..6

3.1 General procedure for the synthesis of the ester derivative-β-carboline **14** and **15**.[2]..6

3.2 Synthesis of racemic **16** by reduction of the ketone-derived **14**..6

3.3 Synthesis of **17** by Pictet-Spengler condensation and $KMnO_4$ oxidation[5]..7

3.4 General procedure for the synthesis of acids **6**, **8**-**11**.[7]..7

3.5 General procedure for the synthesis of the final β-carboline amide derivatives (**6**, **8**-**11**)**a**-**d**..8

3.6 General Procedure for the Synthesis of Amide Standards **12a**, **13a**, **14a**, **15a**, **16a**, **17a**, **18a** and **19a**..8

3.7 Compound characterization..9

4. Biotransformation reactions..16

4.1 Biotransformation reactions..16

4.2 Scaled-up biotransformation..166

5. Analytical data..17

6. Protein Crystallisation, Data Collection and Refinement..23

7. References..22

8. Compound NMR spectra..254

1. General information

Reagents were purchased from Sigma-Aldrich, Alfa Aesar, Acros Organics or Fluorochem and used as received unless otherwise stated. Petroleum ether refers to the fraction of petroleum that is collected at 40-60 °C. Reactions requiring anhydrous conditions were carried out using Schlenk techniques (high vacuum, liquid nitrogen trap on a standard in-house built dual line). Room temperature upper and lower limits are stated as 13-25 °C, but typically 21 °C was recorded. Brine refers to a saturated aqueous solution of NaCl.

Thin layer chromatography (TLC) was carried out using Merck 5554 aluminium backed silica plates (silica gel 60 F254) and spots were visualized using UV light (at 254 nm). Where necessary, plates were stained and heated with one of potassium permanganate, anisaldehyde or vanillin as appropriate. Retention factors (R_f) are reported along with the solvent system used, in parentheses. Flash column chromatography was performed according to the method reported by W. C. Still *et al*[1] using Merck 60 silica gel (particle size 40–63 µm) and a solvent system as reported in the text.

NMR spectra were obtained in the solvent indicated, using a JEOL ECX400 or JEOL ECS400 spectrometer (400MHz, 101 MHz and 162 MHz for ^{1}H, ^{13}C and ^{31}P, respectively), or a Bruker 500 (500 MHz, 126 MHz and 202 MHz for ^{1}H, ^{13}C and ^{31}P, respectively). Chemical shifts are reported in parts per million and were referenced to the residual non-deuterated solvent of the deuterated solvent used ($CHCl_3$ ^{TM}H = 7.26 and ^{TM}C = 77.16 ($CDCl_3$), $(CHD_2)SO(CD_3)$ ^{TM}H = 2.50 and ^{TM}C = 39.52 {$SO(CD_3)_2$}, ^{1}H and ^{13}C, respectively). Spectra were typically run at a temperature of 298 K. All ^{13}C NMR spectra were obtained with ^{1}H decoupling. NMR spectra were processed using MestReNova software (Version 11.0.2-18153, released October 2016). The spectra given below were saved as .emf or .pdf files in MestReNova and inserted directly into a Microsoft Word Document. For the ^{1}H NMR spectra the resolution varies from 0.15 to 0.5 Hz; the coupling constants have been quoted to ± 0.5 Hz in all cases for consistency. ^{1}H and ^{13}C NMR chemical shifts are quoted to 2 decimal places.

Infrared spectra were obtained using a Bruker ALPHA-Platinum FTIR Spectrometer with a platinum-diamond ATR sampling module.

MS spectra were measured using a Bruker Daltronics micrOTOF MS, Agilent series 1200LC with electrospray ionisation (ESI and APCI) or on a Thermo LCQ using electrospray ionisation, with <5 ppm error recorded for all HRMS samples. Mass spectral data is quoted as the *m/z* and mass to charge ratios (*m/z*) are reported in Daltons.

Melting points were recorded using a Stuart digital SMP3 machine.

2. Gene cloning, expression and protein purification

The genes encoding McbA was amplified by PCR using the genomic DNA as template and the following primers:

McbA: 5’-CCAGGGACCAGCAATGGGTTATGCACGTCGTGTTATGGATGGTATTG-3’ (Forward) and 5’-GAGGAGAAGGCGCGTTAGTCGGTGAACCAGGTACGAACTTTAACTTT-3’ (Reverse).

After gel analysis of the PCR, the relevant band was eluted using a PCR Cleanup kit® (Qiagen) and the gene cloned into the pETYSBLIC-3C vector using a ligation-independent cloning (LIC) procedure which has been described previously.[2] Recombinant plasmid containing the McbA gene were then used to transform *E. coli* XL10-Gold Ultracompetent cells (Novagen). Small cultures of transformants yielded plasmids using standard miniprep techniques that were submitted for sequencing to confirm the integrity of the gene. Recombinant vectors were then used to transform *E. coli* BL21 (DE3) cells, using 30 μg mL^{-1} kanamycin as antibiotic marker on Luria-Bertani (LB) agar. One colony produced overnight was then used to inoculate 5 mL of LB broth containing 30 μg mL^{-1} kanamycin, which was then grown for 18 h at 37°C with shaking at 180 r.p.m. This starter culture was then used to inoculate a 500 mL culture of LB broth containing 30 μg mL^{-1} kanamycin and the cultures was grown until the optical density (OD_{600}) had reached 0.6. Expression of the corresponding protein were then induced by the addition of 1 mM isopropyl β-D-1-thiogalactopyranoside (IPTG), after which the culture was incubated at 16 °C in an orbital shaker at 180 r.p.m. for approximately 18 h. Cells were then harvested by centrifugation for 15 min at 4225 *g* in a Sorvall GS3 rotor in a Sorvall RC5B Plus centrifuge and subsequently resuspended in 50 mL 50 mM KP_i buffer pH 7.5 containing 5 % glycerol (w/ v) and 300 mM NaCl. Cells were sonicated for 3 x 30 s bursts at 4 °C with 1 min intervals and the soluble and insoluble fractions separated by centrifugation for 30 min at 26,892 *g* in a Sorvall SS34 rotor. The clear supernatants were loaded onto a 5 mL His-Trap™ Chelating HP nickel column. After washing with 10 column volumes of buffer 50 mM KP_i buffer pH 7.5 containing 5 % glycerol (w/v) and 300 mM NaCl, McbA was eluted with a gradient of 0–500 mM imidazole over 20 column volumes. Column fractions containing the protein, as determined by SDS-PAGE analysis, were pooled and then concentrated using a 30 kDa cut-off Centricon® filter membrane. The concentrated enzymes were then loaded onto a pre-equilibrated S75 Superdex™ 16/60 gel filtration column, and eluted with 120 mL of the buffer, at a flow rate of 1 mL min^{-1}. Fractions containing pure McbA, as determined by SDS-PAGE analysis were pooled and stored at 4 °C.

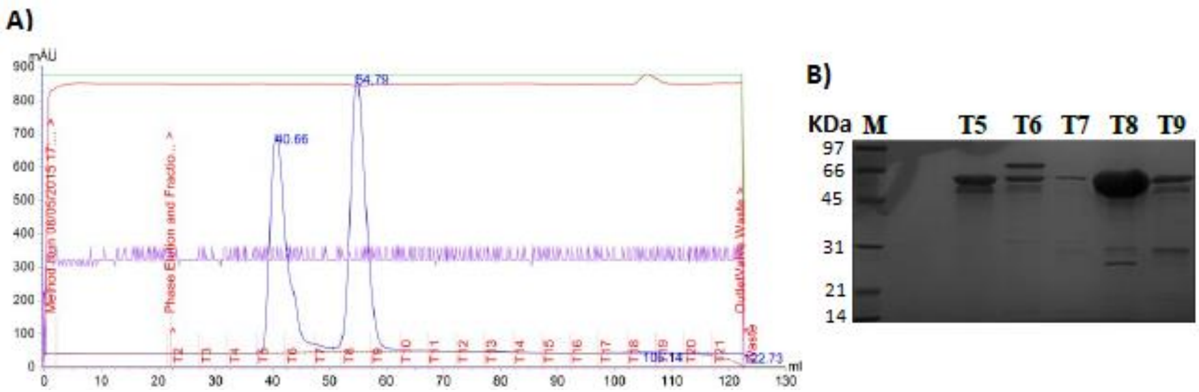

Figure S1. A) Size-exclusion chromatogram of McbA on a S75 Superdex™ 16/60 gel filtration column; **B**) SDS-PAGE showing purification of McbA. **M** = low molecular weight markers (BioRad); Lanes marked **T5** to **T9** correspond to gel filtration column fractions shown in the chromatogram.

2.1 Analytical Size Exclusion Chromatography for Molecular Mass Determination

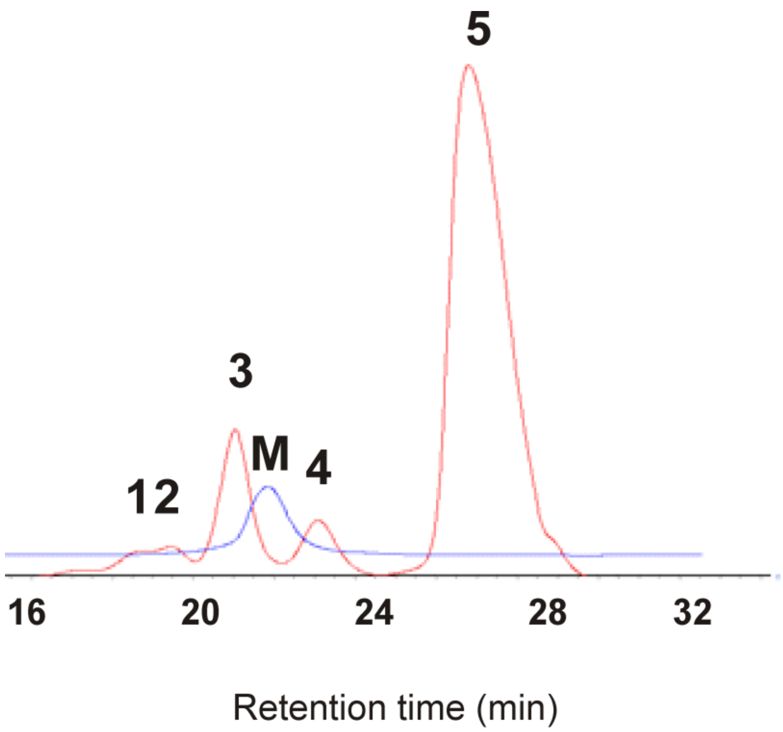

Figure S2. Analytical gel filtration analysis of pure McbA. **Standards: Red curve. 1**: ADH (Approximate Molecular Weight (MW) 15 kDa), 17.0 min retention time (RT); **2**: BSA (MW 66 kDa) 19.1 min RT; **3**: OvA (MW 44.3 kDa) 21.0 min RT; **4:** DNAaseI (MW 30 KDa) 23.1 min RT; **5**: Lysozyme (MW 14.3 kDa) 27.9 min RT. **McbA: Blue curve: M**: (MW 53 kDa) 22.0 min RT.

Conditions: Column: Superdex 200 10/300; Flow rate: 0.75 mL min^{-1}; Eluent: 50 mM Tris HCl pH 7.5, 300 mM NaCl; 0.5 - 1 mg mL^{-1} of protein concentration was loaded in each case, in a loading volume of between 200- 450 μL.

3. Synthesis of Substrates and Product Standards

3.1 General procedure for the synthesis of the β-carboline ester derivates 20 and 21.[2]

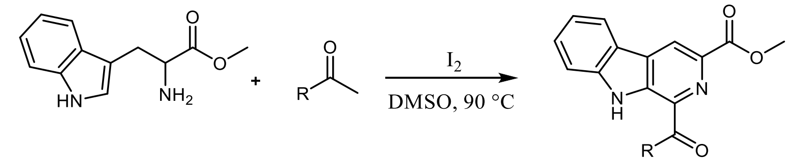

20, R = CH_3, **21**, R = C_6H_5 **20, 21**

Acetone (66 μL, 0.90 mmol) or acetophenone (54 μL, 0.458 mmol) and iodine (115 mg, 0.458 mmol) were added to 2 mL of DMSO and the resulting solution was heated at 90 °C for 1 h. Tryptophan methyl ester (116 mg, 0.458 mmol) was added and the solution was stirred at same temperature for 2-3 h till completion of reaction (monitored by TLC, EtOAc:hexane 1:2). The reaction mixture was then cooled to room temperature followed by addition of water (25 mL) and extracted with EtOAc (2 x 25 mL). The extract was washed with 10% $Na_2S_2O_3$, dried over Na_2SO_4, filtered and the solvent evaporated under reduced pressure. The residue was purified by chromatography on a silica gel using acetone: DCM (0.1: 9.9) as eluent to give the desired product **(20, 21)** as a yellow solid. **20** (72% yield isolated), **21** (56% yield isolated). Compounds **20** and **21** exhibited physical and spectral properties in accordance with those reported.[3]

3.2 Synthesis of racemic 22 by reduction of the ketone-derived 20

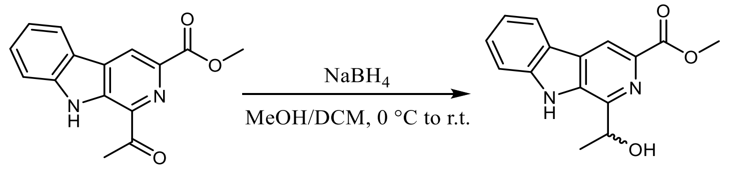

20 ***rac*-22**

$NaBH_4$ (34 mg, 0.90 mmol) was added carefully to a solution of β-carboline derivative **20** (185 mg, 0.69 mmol**)** in a mixture of methanol and $CHCl_2$ (10 mL, 1:1) in an ice bath. The reaction was stirred at 0 °C for 1 h and then at room temperature for 2-3 h until completion of the reaction (monitored by TLC, EtOAc, 5% $v.v^{-1}$ MeOH). The reaction was quenched with water (5 mL) and extracted with $CHCl_2$ (2 x 20 mL). The organic phases were dried over Na_2SO_4, filtered and the solvent evaporated under reduced pressure obtaining, after purification by flask chromatography, the desired product ***rac*-22** as pale yellow solid (87% isolated yield). Compound ***rac*-22**[4] exhibited physical and spectral properties in accordance with those reported.

3.3 Synthesis of 23 by Pictet-Spengler condensation and $KMnO_4$ oxidation[5]

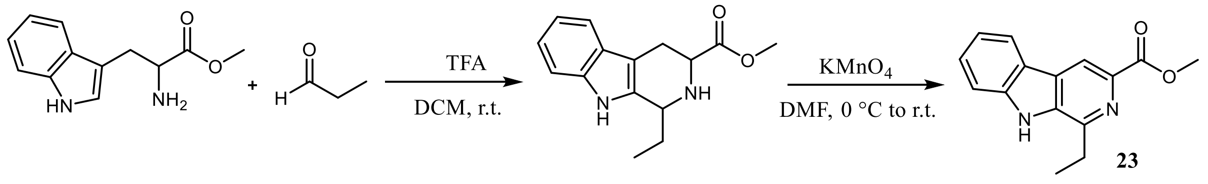

Tryptophan methyl ester (0.5 g; 2.1 mmol), and propionaldehyde (1.2 equiv., 180 μL, 2.5 mmol) were dissolved in DCM (20 mL) and trifluoro acetic acid (1.3 equiv., 190 μL, 2.5 mmol) was added at room temperature. The reaction was stirred overnight and was then quenched with saturated sodium bi-carbonate and extracted with DCM (2 x 20 mL). The organic phases were dried over Na_2SO_4 $_{sat}$, filtered and the solvent evaporated under reduced pressure. The crude product was used in the next step without further purification.[6]

$KMnO_4$ (2 equiv., 660 mg, 4.2 mmol) was added carefully to a cooled stirring solution of the previous crude reaction product in DMF (10 mL) and then the resulting mixture was stirred for 24 h at room temperature. The suspension was filtered over Celite and concentrated under vacuum, obtaining after purification by flask chromatography the desired product **23** as a pale yellow solid (67 % yield isolated). Compound **23**[6] exhibited physical and spectral properties in accordance with those reported.

3.4 General procedure for the synthesis of acids 6, 8-11.[7]

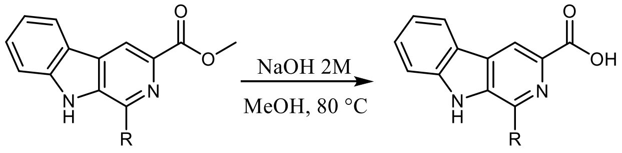

14-18 **(6, 8-11)**

20 (6), R = CH_3, **21 (9)**, R = C_6H_5, **22 (8)**, R = $C(OH)CH_3$, **23 (10)**, **R** = CH_2CH_3, **24 (11)**, **R** = H

A solution of ester derivated-β-carboline **20-24** (113-165 mg, 0.5 mmol) in a mixture of NaOH 2M and methanol (10 mL, 2:1) was stirred at 80 °C for 1-2 h until completion of reaction. After completion, the methanol was eliminated under reduced pressure and then 2M HCl was added, adjusting the pH to 5-6. At this point, a yellow solid precipitated and after cooling the flask in an ice bath the solid was filtered, and then washed with cold water (5 mL) and diethyl ether (2 x 5 mL), obtaining the desired products as pale yellow solids (47-74% isolated yield). Compounds **6**[8], **8**[9], **9**[8], **10**[10] and **11**[11] exhibited physical and spectral properties in accordance with those reported.

3.5 General procedure for the synthesis of the final β-carboline amide derivatives (6, 8-11)a-d

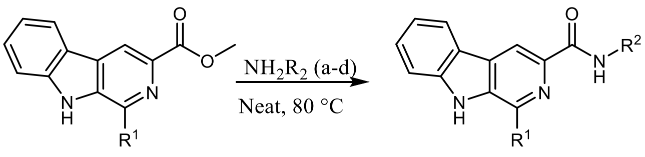

20-24 **(6, 8-11)a-d**

Protocol A) The ester derivative **20-24** (50 mg, 0.2 mmol) was treated with an excess of the respective amine **a-d** (20 equiv.) under neat conditions (the tryptamine **b** was dissolved in DMSO) at 90 °C overnight. After completion of reaction (monitored by TLC) the reaction was cooled to room temperature and extracted with EtOAc (3 x 5 mL) and water (5 mL). The organic extract was dried over $MgSO_4$ and concentrated under vacuum. The crude reaction product was purified by flash chromatography affording the final amide derivatives.

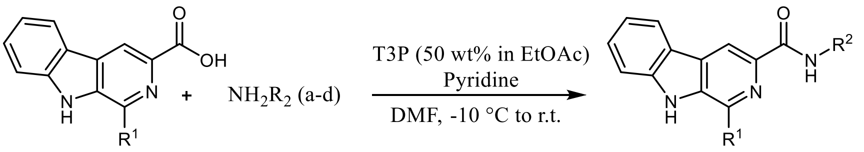

6, 8-11 **(6, 8-11)a-d**

Protocol B)[12] The acid derivative **6, 8-11** (25 mg, 0.1 mmol), amine **a-d** (0.11 mmol, 1.1 equiv.), pyridine (0.5 mmol, 5 equiv.) were added to dry DMF (1 mL). The mixture was cooled to -10 °C and T3P solution (50 wt% in EtOAc, 0.2 mmol, 2 equiv.) was added carefully. The solution was stirred at -10 °C for 10 min and then 1 h at room temperature. The reaction was then cooled to 0 °C, quenched with water (4 mL) and stirred for 5 min. HCl 0.2 N (2 mL) was then added and the reaction was extracted with EtOAc (3 x 10 mL). The organic phases were collected and washed with HCl 0.2 N, NaOH 0.2 N and brine, dried over Na_2SO_4 $_{sat.}$, filtered and evaporated under reduced pressure. The crude reaction product was then purified by flash chromatography, affording the final amide derivatives.

3.6 General Procedure for the Synthesis of Amide Standards 12a, 13a, 14a, 15a, 16a, 17a, 18a and 19a

The acid derivative **18** (100 mg, 0.58 mmol, 1eq.), 2-Phenylethylamine **a** (81 µL, 0.64 mmol, 1.1 equiv.), and Et_3N (404 µL, 2.9 mmol, 5 equiv.), were added to DMF (10 mL). T3P solution (50 wt% in EtOAc, 345 µL, 1.2 mmol, 2 equiv.) was added carefully. The solution was stirred at room temperature for 24 h. The reaction was then quenched with water (5 mL) and stirred for 5 min. HCl 0.2 N (10 mL) was then added and the reaction was extracted with EtOAc (3 x 20 mL). The organic phases were collected and washed with HCl 0.2 N, NaOH 0.2 N and brine, dried over $MgSO_4$ sat., filtered and evaporated under reduced pressure. The amide products were then re-crystallised with Et_2O to afford the final amide derivatives.

3.7 Compound characterization

Data for compounds **6a**[13], **6b**[13], **14a**[14], **15a**[15], **16a**[16], **18a**[17], and **19a**[18] were in accord with those previously described.

1-Acetyl-*N*-(1-phenylethylamine)-*9H*-β-carboline-3-carboxamide (6c)

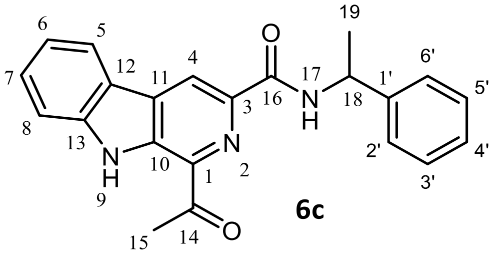

6c

White solid: **mp** 205-208 °C; **Rf** = 0.40 (EtOAc: P.E. 1:2); **IR** (thin film, cm^{-1}): 3382, 3030, 2923, 1662, 1516, 1492, 1463, 1448, 1332, 1248, 1203, 1181, 1150, 755, 743, 698, 679, 623, 599, 571, 541, 506, 429; **^{1}H NMR (400 MHz, $CDCl_3$)** δ 1.71 (d, *J* = 6.9 Hz, 3H, H_{19}), 2.90 (s, 3H, H_{15}), 5.44 (q, *J* = 6.9 Hz, 1H, H_{18}), 7.30 (t, *J* = 7.3 Hz, 1H, $H_{4'}$), 7.44-7.36 (m, 3H, $H_{3'+5'+6}$) 7.48 (d, *J* = 7.6 Hz 2H, $H_{2'+6'}$), 7.65-7.59 (m, 2H, H_{7+8}), 8.20 (d, *J* = 7.9 Hz, 1H, H_5), 8.31 (d, *J* = 8.3 Hz 1H, H_{17}), 9.11 (s, 1H, H_4), 10.42 (s, 1H, H_9); **^{13}C NMR (400 MHz, $CDCl_3$)** δ 22.50 (CH_3, C_{19}), 25.85 (CH_3, C_{15}), 48.97 (CH, C_{18}),112.32 (CH, C_8), 118.67 (CH, C_4), 121.15 (C, C_{12}), 121.67 (CH, C_5), 122.43 (CH, C_6), 126.23 (2CH, $C_{3'+5'}$), 127.50 (CH, $C_{4'}$), 128.91 (2CH, $C_{2'+6'}$), 129.88 (CH, C_7), 132.77 (C, $C_{1'}$), 133.63 (C, C_1), 136.47 (C, C_{11}), 139.34 (C, C_{10}), 141.66 (C, C_3), 143.65 (C, C_{13}), 163.94 (C, C_{16}), 202.32 (C, C_{14}); **HRMS** (ESI+, m/z): calculated for $(C_{22}H_{20}N_3O_2)^+$ $[(M+H)^+]$: 358.1550; found: 358.1553.

1-Acetyl-*N*-(3-phenyl-1-methylpropylamine)-*9H*-β-carboline-3-carboxamide (6d)

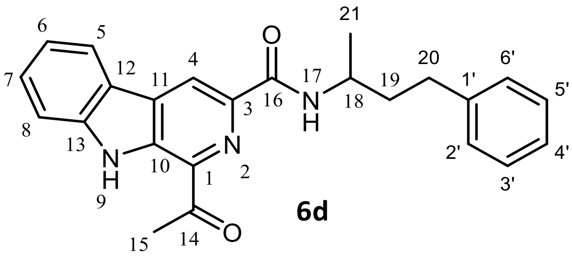

6d

White solid: **mp** 178-180 °C; **Rf** = 0.40 (EtOAc: P.E. 1:2); **IR** (thin film, cm^{-1}): 3344, 3059, 3024, 2959, 2934, 2919, 2853, 1652, 1593, 1523, 1494, 1461, 1450, 1360, 1331, 1286, 1249, 1209, 1181, 1150, 1060, 966, 755, 739, 715, 694, 677, 629, 585, 562, 507, 491, 481, 460, 427; **^{1}H NMR (400 MHz, $CDCl_3$)** δ 1.37 (d, *J* = 6.7 Hz, 3H, H_{21}), 1.98-1.95 (m, 2H, H_{19}), 2.76 (t, *J* = 8.0 Hz, 2H, H_{20}), 2.89 (s, 3H, H_{15}), 4.37-4.29 (m, 1H, H_{18}), 7.13 (t, *J* = 6.9 Hz, 1H, $H_{4'}$), 7.26-7.19 (m, 4H, $H_{2'+3'+5'+6'}$) 7.36 (t, *J* = 7.3 Hz 1H, H_6), 7.62-7.57 (m, 2H, H_{7+8}), 7.85 (d, *J* = 8.7 Hz, 1H, H_5), 8.20 (d, *J* = 7.9 Hz 1H, H_{17}), 9.10 (s, 1H, H_4), 10.40 (s, 1H, H_9); **^{13}C NMR (400 MHz,**

$CDCl_3$) δ 21.42 (CH_3, C_{21}), 25.85 (CH_3, C_{15}), 32.79 (CH_2, C_{19}), 39.15 (CH_2, C_{20}),45.39 (CH, C_{18}), 112.31 (CH, C_8), 118.59 (CH, C_4), 121.18 (C, C_{12}), 121.66 (CH, C_5), 122.44 (CH, C_6), 126.00 (CH, $C_{4'}$), 128.49 (2CH, $C_{3'+5'}$), 128.55 (2CH, $C_{2'+5'}$), 129.86 (CH, C_7), 132.79 (C, $C_{1'}$), 133.59 (C, C_1), 136.44 (C, C_{11}), 139.52 (C, C_{10}), 141.67 (C, C_3), 141.93 (C, C_{13}), 164.13 (C, C_{16}), 202.32 (C, C_{14});

1-(1-Hydroxyethyl)-*N*-(2-phenylethylamine)-*9H*-β-carboline-3-carboxamide (8a)

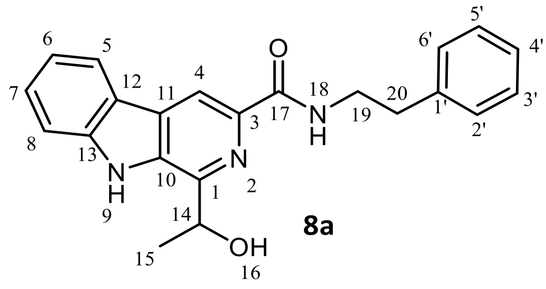

8a

White solid: **mp** 188-191 °C; **Rf** = 0.30 (EtOAc: P.E. 1:1); **IR** (thin film, cm^{-1}): 3368, 3232, 2979, 2928, 1638, 1594, 1565, 1536, 1495, 1467, 1452, 1381, 1347, 1321, 1293, 1254, 1164, 1146, 1096, 1049, 1026, 932, 796, 782, 775, 744, 724, 710, 694, 676, 618, 585, 533, 497, 467, 431; **^{1}H NMR (400 MHz, DMSO-*d*$_6$)** δ 1.58 (d, *J* = 6.5 Hz, 3H, H_{15}), 2.91 (t, *J* = 8.0 Hz, 2H, H_{20}), 3.61(q, *J* = 8.0 Hz, 2H, H_{19}), 5.29 (q, *J* = 6.3 Hz, 1H, H_{14}), 5.81 (d, *J* = 6.5 Hz, 1H, OH_{16}), 7.33-7.10 (m, 6H, H_6+$H_{2'-6'}$), 7.57 (t, *J* = 7.6 Hz, 1H, H_7), 7.73 (d, *J* = 8.3 Hz, 1H, H_8), 8.35 (d, *J* = 7.8 Hz, 1H, H_5), 8.74 (s, 1H, H_4), 8.92 (t, *J* = 8.1 Hz, 1H, H_{18}), 11.72 (s, 1H, H_9); **^{13}C NMR (400 MHz, DMSO-*d*$_6$)** δ 23.36 (CH_3, C_{15}), 35.55 (CH_2, C_{20}), 40.44 (CH_2, C_{19}), 68.23 (CH, C_{14}), 112.59 (CH, C_8), 112.70 (CH, C_4), 119.84 (CH, C_5), 120.99 (C, C_{12}), 121.87 (CH, C_6), 126.16 (CH, C_7), 128.34 (CH, $C_{4'}$), 128.42 (2CH, $C_{2',6'}$), 128.70 (CH, $C_{3',5'}$), 128.80 (C, $C_{1'}$) , 133.35 (C, C_1), 138.30 (C, C_{11}), 139.57(C, C_{10}), 141.04(C, C_3), 147.01 (C, C_{13}), 164.68 (C, C_{17}); **HRMS** (ESI+, m/z): calculated for $(C_{22}H_{22}N_3O_2)^+$ [$(M+H)^+$]: 360.1707; found: 360.1698.

1-(1-Hydroxyethyl)-*N*-(tryptamine)-*9H*-β-carboline-3-carboxamide (8b)

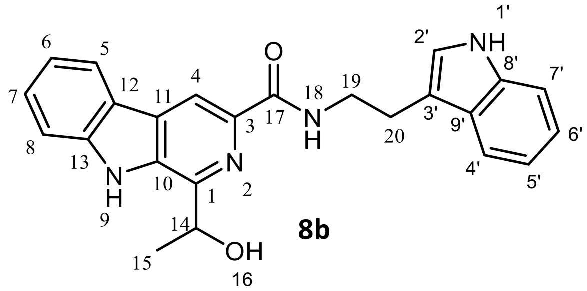

8b

Yellow solid: **mp** 207-210 °C; **Rf** = 0.20 (EtOAc: P.E. 1:1); **IR** (thin film, cm^{-1}): 3355, 2962, 2922, 1722, 1640, 1625, 1593, 1537, 1497, 1455, 1372, 1349, 1257, 1087, 1012, 942, 903, 869, 793, 739, 681, 661, 600, 587, 560, 502, 463, 435, 422; **^{1}H NMR (400 MHz, DMSO-*d*$_6$)** δ

1.52 (d, J = 6.5 Hz, 3H, H_{15}), 2.98 (t, J = 7.4 Hz, 2H, H_{20}), 3.63 (q, J = 7.4 Hz, 2H, H_{19}), 5.23 (q, J = 6.5 Hz, 1H, H_{14}), 5.76 (d, J = 6.4 Hz, 1H, H_{16}), 6.95 (t, J = 7.6 Hz, 1H, $H_{5'}$), 7.03 (t, J = 7.6 Hz, 1H, $H_{6'}$), 7.25-7.05 (m, 3H, $H_{6+2'}$), 7.31 (d, J = 8.0 Hz, 1H, $H_{7'}$), 7.52 (t, J = 8.2 Hz, 1H, H_7), 7.62 (d, J = 7.8 Hz,1H, $H_{4'}$), 7.67 (d, J = 8.2 Hz, 1H, H_8), 8.31 (d, J = 7.9 Hz, 1H, H_5), 8.70 (s, 1H, H_4), 8.92 (t, J = 6.0 Hz, 1H, H_{18}), 10.82 (s, 1H, $H_{1'}$), 11.66 (s, 1H, H_9); **^{13}C NMR (400 MHz, DMSO-*d*$_6$)** δ 23.38 (CH_3, C_{15}), 25.55 (CH_2, C_{20}), 59.82 (CH_2, C_{19}), 68.33 (CH, C_{14}), 111.42 (CH, $C_{7'}$), 111.94 (C, $C_{8'}$), 112.61 (C, $C_{3'}$), 112.71 (CH, C_8), 118.29 (CH, C_4), 118.49 (CH, $C_{4'}$), 119.86 (CH, $C_{5'}$), 120.99 (CH, $C_{6'}$), 121.90 (CH, C_5), 122.70 (CH, C_6), 125.37 (C, C_{12}), 127.31 (C, $C_{9'}$), 128.81 (CH, $C_{2'}$), 128.95 (CH, C_7), 133.31 (C, C_1), 136.33 (C, C_{11}), 138.41 (C, C_{10}), 141.04 (C, C_3), 147.05 (C, C_{13}), 164.68 (C, C_{17}); **HRMS** (ESI+, m/z): calculated for $(C_{24}H_{23}N_4O_2)^+$ $[(M+H)^+]$: 399.1816; found: 399.1804.

1-(1-Hydroxyethyl)-*N*-(3-phenyl-1-methylpropylamine)*9H*-β-carboline-3-carboxamide (8d)

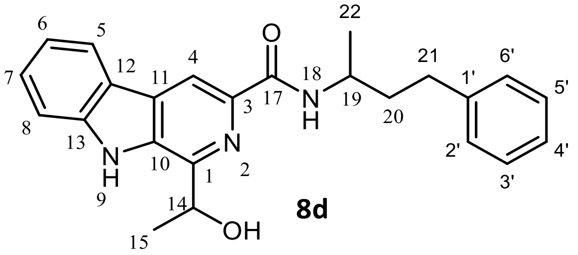

8d

White solid: **mp** 173-176 °C; **Rf =** 0.33 (EtOAc: P.E.2:1); **IR** (thin film, cm^{-1}): 3273, 2964, 2926, 2853,1644, 1625, 1597, 1566, 1530, 1495, 1466, 1447, 1357, 1249, 1165, 1141, 1077, 1060, 1030, 1016, 969, 902, 881, 854, 794, 741, 697, 621, 574, 477, 434, 407; **^{1}H NMR** (400 MHz, **CDCl$_3$**) δ 1.33 (d, J = 6.7, 4.5 Hz, 3H, H_{22}), 1.73 (d, J = 6.7 Hz, 3H, H_{15}), 1.96-1.87 (m, 2H, H_{21}), 2.74 (dt, J = 10.2, 8.1 Hz, 2H, H_{20}), 4.33-4.26 (m, 1H, H_{19}), 5.42-5.37 (m, 1H, H_{14}), 7.25-7.11 (m, 6H, $H_{Ar'+5}$), 7.53-7.50 (m, 1H, H_7), 7.92 (d, J = 9.0 Hz, 1H, H_8), 8.01 (dd, J = 11.0, 7.8 Hz, 1H, H_6), 8.65 (d, J = 13.9 Hz, 1H, H_{18}), 9.49 (s, 1H, H_4), 11.67 (s, NH, H_9); **^{13}C NMR** (101 MHz, **CDCl$_3$**) δ 21.34 (CH, C_{22}), 23.18 (CH, C_{15}), 32.75 (CH, C_{21}), 39.06 (CH, C_{20}), 45.23 (CH, C_{19}), 72.19 (CH, C_{14}), 111.76 (CH, C_8), 113.48 (CH, C_4), 120.65 (CH, C_5), 121.73 (CH, C_{12}), 121.97 (CH, C_6), 125.97 (CH, C_7), 128.49 (CH, $C_{2',6'}$), 128.54 (CH, $C_{3',5'}$), 128.80 (CH, $C_{4'}$), 130.35 (CH, $C_{1'}$), 134.27 (CH, C_1), 138.86 (CH, C_{11}), 140.50 (CH, C_{10}), 142.03 (CH, C_3), 145.54 (CH, C_{13}), 165.12 (CH, C_{17}); **HRMS** (ESI+, m/z): calculated for $(C_{24}H_{25}N_3O_3)^+$ $[(M+H)^+]$: 388.2020; found: 388.2005.

1-(Benzoyl)-*N*-(2-phenylethylamine)-*9H*-β-carboline-3-carboxamide (9a)

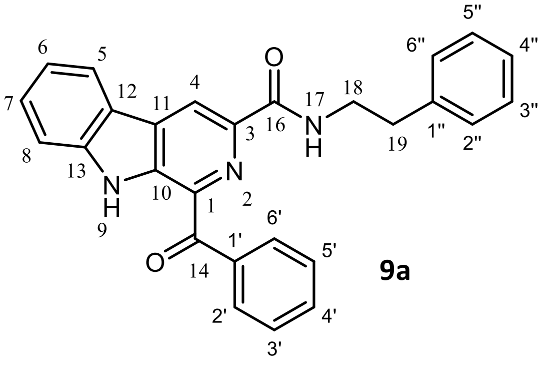

9a

Yellow solid: **mp** 202-204 °C; **Rf** = 0.40 (EtOAc: P.E. 1:2); **IR** (thin film, cm^{-1}): 3406, 3226, 3058, 2916, 2851, 1657, 1646, 1621, 1522, 1491, 1453, 1365, 1346, 1333, 1290, 1256, 1242, 1216, 1205, 1177, 1143, 1110, 1085, 1071, 1028, 1011, 993, 974, 868, 852, 798, 786, 739, 698, 684, 660, 635, 615, 588, 567, 524, 508, 489, 437; **^{1}H NMR** (400 MHz, $CDCl_3$) δ 2.96 (t, *J* = 6.9 Hz, 2H, H_{19}), 3.78 (q, *J* = 6.8 Hz, 2H, H_{18}), 7.18-7.64 (m, 12H, H_{Ar+6+7}), 7.66 (t, *J* = 6.5 Hz, 1H, H_{17}), 8.10 (d, *J* = 7.5 Hz, 1H, H_8), 8.27 (d, *J* = 7.9 Hz, 1H, H_5), 9.14 (s, 1H, H_4), 10.53 (s, 1H, H_9). **^{13}C NMR** (400 MHz, $CDCl_3$) δ 36.02 (CH_2, C_{19}), 40.99 (CH_2, C_{18}), 112.37 (CH, C_8), 118.00 (CH, C_4), 121.48 (C, C_{12}), 121.78 (CH, C_5), 122.49 (CH, C_6), 126.64 (CH, $C_{4''}$), 128.17 (2CH, $C_{3'',5''}$), 128.74 (2CH, $C_{2'',6''}$), 128.90 (2CH, $C_{3',5'}$), 129.96 (CH, $C_{4'}$), 130.98 (2CH, $C_{2',6'}$), 132.68 (CH, C_7), 132.99 (C, $C_{1'}$), 133.58 (C, $C_{1''}$), 137.42 (C, C_1), 138.32 (C, C_{11}), 139.19 (C, C_3), 139.20 (C, C_{10}), 141.58 (C, C_{13}), 164.94 (C, C_{15}), 195.22 (C, C_{14}); **HRMS** (ESI+, m/z): calculated for $(C_{27}H_{21}N_3NaO_2)^+$ $[(M+Na)^+]$: 442.1526; found: 442.1513.

1-(Ethyl)-*N*-(2-ethylamine)-*9H*-β-carboline-3-carboxamide (10a)

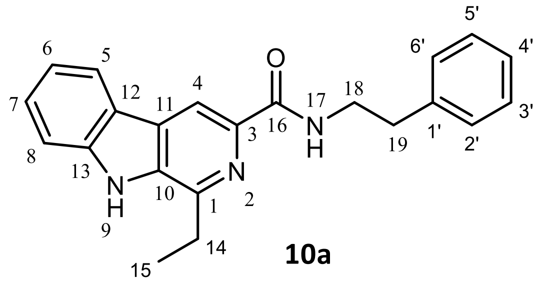

10a

White solid: **mp** 177-179 °C; **Rf** = 0.38 (EtOAc: *n*-Hex. 1:1); **IR** (thin film, cm^{-1}): 3371, 3144, 3085, 3059, 3025, 2958, 2928, 2872, 1644, 1624, 1595, 1563, 1530, 1498, 1464, 1453, 1440, 1362, 1341, 1322, 1291, 1248, 1144, 1096, 1083, 1045, 1030, 1012, 975, 928, 892, 866, 840, 780, 735, 696, 619, 568, 489, 468, 429; **^{1}H NMR** (400 MHz, **DMSO-d6**) δ 1.41 (t, *J* = 7.5 Hz, 3H, H_{15}), 2.98 (t, *J* = 7.1 Hz, 2H, H_{19}), 3.14 (q, *J* = 7.5 Hz, 2H, H_{14}), 3.82 (q, *J* = 7.2 Hz, 2H, H_{18}), 7.22-7.30 (m, 6H, H_6+$H_{2'-6'}$), 7.50 (t, *J* = 7.9 Hz, 1H, H_7), 7.58 (d, *J* = 8.1 Hz, 1H, H_8), 8.06 (d, *J* = 7.9 Hz, 1H, H_5), 8.47 (t, *J* = 7.1 Hz, 1H, H_{17}), 8.78 (s, 1H, H_4), 9.79 (s, 1H, H_9); **^{13}C NMR** (400 MHz, **$CDCl_3$**) δ 12.02 (CH_3, C_{15}), 26.81 (CH_2, C_{14}), 36.28 (CH_2, C_{19}), 40.84 (CH_2, C_{18}), 111.78

(CH, C_8), 112.67 (CH, C_4), 120.93 (CH, C_6), 122.27 (C, C_{12}), 122.65 (CH, C_5), 126.56 (CH, C_7), 128.65 (C, $C_{4'}$), 128.74 (2CH, $C_{3',5'}$), 129.10 (C, $C_{1'}$), 135.34 (2CH, $C_{2',6'}$), 139.44 (C, C_1), 139.99 (C, C_{11}), 140.34 (C, C_3), 144.77 (C, C_{10}), 145.27 (C, C_{13}), 166.22 (C, C_{16}); **HRMS** (ESI+, m/z): calculated for $(C_{22}H_{22}N_3O)^+$ [$(M+H)^+$]: 344.1757; found: 344.1760.

1-(Ethyl)-*N*-(tryptamine)-*9H*-β-carboline-3-carboxamide (10b)

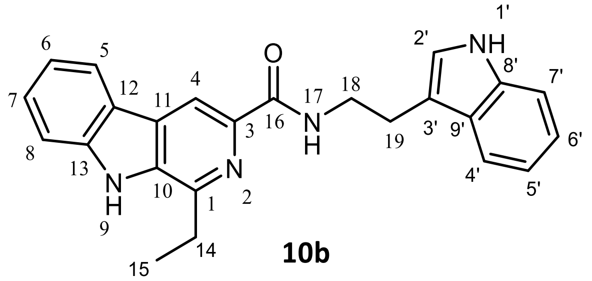

10b

Yellow solid: **mp** 264-267 °C; **Rf** = 0.30 (EtOAc: P.E.1:1); **IR** (thin film, cm^{-1}): 3414, 3364, 2963, 2927, 2850, 1639, 1535, 1497, 1450, 1432, 1354, 1336, 1261, 1248, 1218, 1096, 1077, 803, 743, 697, 681, 639, 616, 597, 585, 559, 520, 488, 457, 433, 422; **^{1}H NMR** (400 MHz, **DMSO-*d*$_6$**) δ 1.39 (t, *J* = 7.5 Hz, 3H, H_{15}), 3.02 (t, *J* = 7.4 Hz, 2H, H_{19}), 3.16 (q, *J* = 7.4 Hz, 2H, H_{14}), 3.68 (q, *J* = 7.5 Hz, 2H, H_{18}), 6.99 (t, *J* = 7.4 Hz, 1H, $H_{5'}$), 7.08 (t, *J* = 7.4 Hz, 1H, $H_{6'}$) 7.29-7.22 (m, 2H, $H_{6+2'}$), 7.37 (d, *J* = 7.5 Hz, 1H, $H_{7'}$), 7.57 (t, *J* = 7.5 Hz, 1H, H_7) 7.68-7.55 (m, 2H, $H_{8+4'}$), 8.34 (d, *J* = 7.8 Hz, 1H, H_5), 8.69-8.66 (m, 2H, H_{17+4}), 10.88 (s, 1H, $H_{1'}$), 11.93 (s, 1H, H_9); **^{13}C NMR** (400 MHz, **DMSO-*d*$_6$**) δ 12.36 (CH_3, C_{15}), 25.45 (CH_2, C_{14}), 26.45 (CH_2, C_{19}), 39.94 (CH_2, C_{18}), 111.40 (CH, $C_{7'}$), 111.86 (C, $C_{3'}$), 111.92 (CH, C_8), 112.20 (CH, C_4), 118.27 (CH, $C_{4'}$), 118.51 (CH, $C_{5'}$), 119.88 (CH, $C_{6'}$), 121.00 (CH, C_5), 121.49 (C, $C_{8'}$), 122.08 (CH, C_5), 122.75 (CH, $C_{2'}$), 127.28 (C, C_{12}), 127.70 (C, $C_{9'}$), 128.27 (CH, C_7), 135.11 (C, C_1), 136.35 (C, C_{11}), 139.13 (C, C_{10}), 140.82 (C, C_3), 145.45 (C, C_{13}), 164.76 (C, C_{16}); **HRMS** (ESI+, m/z): calculated for $(C_{24}H_{23}N_4O)^+$ [$(M+H)^+$]: 383.1866; found: 383.1870.

***N*-(2-Phenylethylamine)-*9H*-β-carboline-3-carboxamide (11a)**

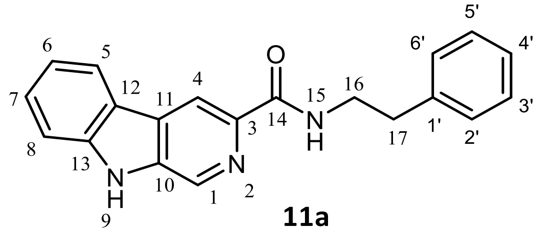

11a

White solid: **mp** 206-209 °C; **Rf** = 0.38 (EtOAc: P.E. 1:1); **IR** (thin film, cm^{-1}): 3367, 3148, 2928, 1647, 1623, 1593, 1531, 1495, 1460, 1452, 1439, 1333, 1315, 1262, 1246, 1200, 1017, 751, 732, 712, 699, 668, 631, 592, 572, 495, 487, 421; **^{1}H NMR** (400 MHz, **DMSO-*d*$_6$**) δ 2.91

(t, J = 7.4 Hz, 2H, H_{17}), 3.62 (q, J = 6.4 Hz, 2H, H_{16}), 7.31-7.20 (m, 6H, H_6+$H_{2'-6'}$), 7.59 (t, J = 7.9 Hz, 1H, H_7), 7.66 (d, J = 8.2 Hz, 1H, H_8), 8.39 (d, J = 7.9 Hz, 1H, H_5), 8.75 (t, J = 6.0 Hz, 1H, H_{15}), 8.86 (s, 1H, H_1), 8.89 (s, *1*H, H_4), 11.96 (s, 1H, H_9);**^{13}C NMR** (400 MHz, **DMSO-*d*$_6$**) δ 35.43 (CH_2, C_{17}), 40.35 (CH_2, C_{18}), 112.29 (CH, C_8), 113.94 (CH, C_4), 120.00 (CH, C_5), 121.00 (C, C_{12}), 122.27 (CH, C_6), 126.13 (CH, C_7), 128.20 (C, $C_{1'}$), 128.40 (2CH, $C_{3',5'}$), 128.65 (CH, $C_{4'}$), 128.67 (2CH, $C_{2',6'}$), 132.30 (CH, C_1), 137.17 (C, C_{11}), 139.56 (C, C_{10}), 139.75 (C, C_3), 141.08 (C, C_{13}), 164.69 (C, C_{14}); **HRMS** (ESI+, m/z): calculated for $(C_{20}H_{18}N_3O)^+$ [$(M+H)^+$]: 316.1444; found: 316.1438.

***N*-(Tryptamine)-*9H*-β-carboline-3-carboxamide (11b)**

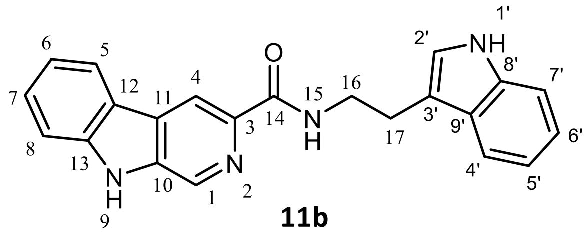

11b

Yellow solid: **mp** 202-204 °C; **Rf** = 0.21 (EtOAc: P.E. 1:1); **IR** (thin film, cm^{-1}): 3405, 3218, 2960, 2922, 2852, 1645, 1623, 1595, 1532, 1500, 1459, 1336, 1310, 1258, 1219, 1197, 1142, 1093, 1051, 1015, 968, 933, 893, 858, 805, 772, 743, 726, 702, 658, 630, 599, 591, 561, 507, 479, 468, 420; **^{1}H NMR (400 MHz, DMSO-*d*$_6$)** δ 2.96 (t, J = 7.4 Hz, 2H, H_{17}), 3.68 (q, J = 7.4 Hz, 2H, H_{16}), 6.94 (t, J = 7.3 Hz, 1H, $H_{5'}$), 7.03 (t, J = 7.3 Hz, 1H, $H_{6'}$) 7.18 (d, J = 2.0 Hz 1H, $H_{2'}$), 7.23-7.31 (m, 2H, $H_{7'+6}$), 7.55 (t, J = 8.2 Hz, 1H, H_7), 7.60-7.62 (m, 2H, $H_{8+4'}$), 8.35 (d, J = 7.7 Hz, 1H, H_5), 8.77 (t, J = 6.0 Hz, 1H, H_{14}), 8.82 (s, 1H, H_1), 8.83 (s, 1H, H_4), 10.80 (s, 1H, $H_{1'}$), 11.91 (s, 1H, H_9); **^{13}C NMR (400 MHz, DMSO-*d*$_6$)** δ 25.53 (CH_2, C_{17}), 39.54 (CH_2, C_{16}), 111.40 (CH, $C_{7'}$), 111.91 (C, $C_{8'}$), 112.25 (C, $C_{3'}$), 112.29 (CH, C_8), 113.94 (CH, C_4), 118.28 (CH, $C_{4'}$), 118.52 (CH, $C_{5'}$), 120.01 (CH, $C_{6'}$), 121.01 (CH, C_5), 122.28 (CH, C_6), 122.63 (CH, $C_{2'}$), 127.32 (C, $C_{9'}$), 128.21 (C, C_{12}), 128.65 (CH, C_7), 132.28 (CH, C_1), 136.32 (C, C_{11}), 137.15 (C, C_{10}), 139.86 (C, C_3), 141.07 (C, C_{13}), 164.67 (C, C_{14}); **HRMS** (ESI+, m/z): calculated for $(C_{22}H_{19}N_4O)^+$ [$(M+H)^+$]: 355.1553; found: 355.1555.

1-(Acetyl)-*N*-(2-Phenylethylamine)-Pyridine-3-carboxamide (12a)

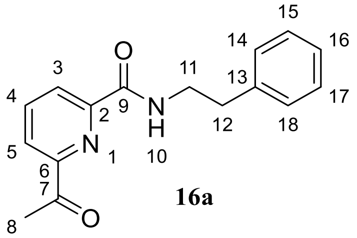

16a

Light brown solid: **mp** 114-116 °C; **Rf** = 0.26 (EtOAc: *n*-Hex. 1:1); **IR** (thin film, cm^{-1}): 3345, 3079, 3027, 2926, 2861, 1698, 1672, 1651, 1543, 1490, 1450, 1417, 1357, 1312, 1298, 1260, 1242, 1200, 1177, 1151, 1107, 1078, 1013, 994, 977, 956, 919, 893, 839, 805, 784, 766, 756, 744, 704, 653, 628, 608, 592, 536, 491, 463, 417; **^{1}H NMR (400 MHz, DMSO-*D_6*)** δ 8.86 (t, *J* = 6.0 Hz, NH), 8.24 (d, *J* = 1.7 Hz, 1H, H_3), 8.17 (t, *J* = 7.7 Hz, 1H, H_4), 8.09 (dd, *J* = 7.6, 1.3 Hz, 1H, H_5), 7.36 – 7.16 (m, 5H, H_{14-18}), 3.58 (q, *J* = 7.2 Hz, 2H, H_{11}), 2.89 (t, *J* = 7.2 Hz, 2H, H_{12}), 2.73 (s, 3H, H_8); **^{13}C NMR (101 MHz, DMSO-*D_6*)** δ 199.15 (C, C_9), 163.16 (C, C_7), 151.81 (C, C_2), 149.52 (C, C_6), 139.33 (C, C_3), 139.30 (C, C_{13}), 128.72 (2C, C_{14+18}), 128.50 (2C, C_{15+17}), 126.27 (C, C_{16}), 125.39 (C, C_5), 123.50 (C, C_4), 40.62 (C, C_{11}), 35.23 (C, C_{12}), 25.81 (C, C_8); **HRMS** (ESI+, m/z): calculated for $(C_{16}H_{16}N_2O_2)^+$ $[(M+H)^+]$: 269.1285; found: 269.1286.

***N*-(2-Phenylethylamine)-Isoquinoline-3-carboxamide (13a)**

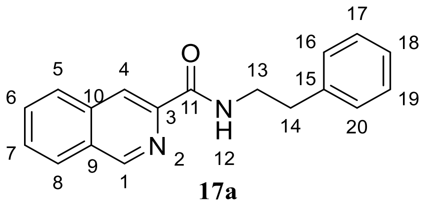

17a

White solid: **mp** 90-92 °C; **Rf** = 0.34 (EtOAc: *n*-Hex. 1:1); **IR** (thin film, cm^{-1}): 3352, 3055, 3028, 2926, 2858, 1650, 1624, 1584, 1518, 1494, 1453, 1432, 1386, 1372, 1335, 1321, 1306, 1293, 1278, 1262, 1229, 1189, 1177, 1145, 1115, 1085, 1046, 1029, 1017, 987, 969, 949, 930, 908, 870, 814, 802, 768, 748, 696, 657, 641, 607, 575, 502, 471, 410; **^{1}H NMR (400 MHz, DMSO-*D_6*)** δ 9.36 (s, 1H, H_4), 8.98 (t, *J* = 5.8 Hz, NH, H_{12}), 8.55 (s, 1H, H_1), 8.24 (dd, *J* = 8.1, 0.7 Hz, 1H, H_5), 8.19 (dd, *J* = 8.1, 0.7 Hz, 1H, H_8) 7.92 – 7.75 (m, 2H, H_{6+7}), 7.35 – 7.17 (m, 5H, H_{16-20}), 3.61 (q, *J* = 7.3, 6.4 Hz, 2H, H_{13}), 2.89 (t, *J* = 7.8 Hz, 1H, H_{14}); **^{13}C NMR (101 MHz, DMSO-*D_6*)** δ 164.00 (C, C_{11}), 151.54 (C, C_4), 143.82 (C, C_3), 139.48 (C, C_9), 135.43 (C, C_{10}), 131.39 (C, C_1), 129.22 (C, C_{15}), 129.11 (C, C_5), 128.67 (C, C_{16+20}), 128.41 (C, C_{17+19}), 128.00 (C, $C_{6/7}$), 127.84 (C, $C_{6/7}$), 126.15 (C, C_{18}), 119.63 (C, C_8), 40.45 (C, C_{13}), 35.26 (C, C_{14}); **HRMS** (ESI+, m/z): calculated for $(C_{18}H_{17}N_2O)^+$ $[(M+H)^+]$: 277.1335; found: 277.1323.

N-(2-Phenylethyl)-Indole-5-Carboxamide (17a)

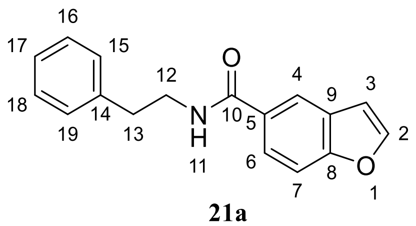

21a

White solid: **mp** 121-123 °C; **Rf** = 0.32 (EtOAc: *n*-Hex. 1:1); **IR** (thin film, cm^{-1}): 3373, 1638, 1542, 1526, 1496, 1454, 1309, 1290, 1261, 1207, 1177, 1115, 1025, 833, 777, 757, 712, 694, 644, 591, 567, 503, 468, 419; **^{1}H NMR (400 MHz, CHLOROFORM-*D*)** δ 7.99 (d, *J* = 1.7 Hz 1H, H_4), 7.67 (d, *J* = 2.2 Hz, 1H, H_2), 7.65 (dd, *J* = 8.6, 1.9 Hz, 1H, H_6), 7.50 (dt, *J* = 8.6, 0.8 Hz, 1H, H_7), 7.36 – 7.25 (m, 5H, H_{15}-H_{19}), 6.84 – 6.76 (m, 1H, H_3), 6.27 (s, NH), 3.75 (q, *J* = 6.7 Hz, 2H, H_{12}), 2.95 (t, *J* = 6.9 Hz, 2H, H_{13}); **^{13}C NMR (101 MHz, CHLOROFORM-*D*)** δ 167.80 (C, C_{10}), 156.58 (C, C_8), 146.33 (C, C_2), 139.07 (C, C_5), 129.90 (C, C_9), 128.93 (2C, C_{15+19}), 128.81 (2C, C_{16+18}), 127.61 (C, C_{14}), 126.67 (C, C_6), 123.33(C, C_4), 120.64 (C, C_{17}), 111.47 (C, C_7), 107.07 (C, C_3), 41.35 (C, C_{12}), 35.84 (C, C_{13}); **HRMS** (ESI+, m/z): calculated for $(C_{17}H_{16}NO_2)^+$ $[(M+H)^+]$: 266.1176; found: 266.1167.

4. Biotransformation reactions

4.1 Biotransformation reactions

Method I: In a 1.5 mL Eppendorf vial, McbA (1 mg mL^{-1}) was added in phosphate buffer (50 μL, 50 mM, pH 7.5, 2 mM ATP, DMSO 4 % $v.v^{-1}$) and mixed with the amine **a-d** (0.6 mM), β-carboline-3-carboxylic acid **6, 8-11**(0.4 mM). Reactions were shaken at 37 °C and 180 r.p.m. for 24 h and stopped by extraction with EtOAc (2 x 250 μL), concentrated and redissolved in 100 μL of MeOH for HPLC analysis.

Method II: In a 1.5 mL Eppendorf vial, McbA (1 mg mL^{-1}) was added in phosphate buffer (100 μL, 50 mM, pH 7.5, 2 mM ATP, DMSO 4 % $v.v^{-1}$) and mixed with the amine **a** (0.6 mM), and acid **12-19** (0.4 mM). Reactions were shaken at 37 °C and 180 r.p.m. for 24 h and stopped by adding 40% MeCN (0.1% Formic Acid). The samples were then filtered for HPLC analysis.

Conversions were determined by comparing peak areas for products against calibration curves determined using each product.

4.2 Scaled-up biotransformations

Into a 250 mL Erlenmeyer flask, a solution of 1-acetyl-3-carboxy-β-carboline **6** (50 mg, 0.196 mmol, 4 mM) in 2 mL of DMSO (4% $v.v^{-1}$ of the total volume), 2-phenylethylamine (38 μL, 1.5 equiv.) and ATP (198 mg, 2 equiv.) were dissolved in phosphate buffer (50 mL, 50 mM, pH 7.5). Then McbA (1 mg mL^{-1}) was added to a final concentration of 1 mg mL^{-1}) and the

reactions were shaken at 37 ^{o}C and 180 rpm. After 24 h the reaction was completed (checked by TLC) the crude was extracted with EtOAc (4 x 30 mL), The organic phases were collected and washed with HCl 0.1 N (50 mL), NaOH 0.1 N (50 mL), and brine (50 mL), dried over Na_2SO_4, filtered and the solvent evaporated under reduced pressure, affording the desired product as a white powder **6a** (67 mg, 85% isolated yield) without any further purification.

The scale up of **17a** was carried out by adding a further 1 mg mL^{-1} McbA and 1 eq. ATP to the reaction after 24h. The reaction was then incubated at 37 °C for a further 24h.

For amides **7a**, **14a**, **16a** and **17a**, only Ni-NTA was performed as the purification step. The imidazole was then dialysed out with 50 mM HEPES, 300 mM NaCl, 10 mM $MgCl_2$, 10% glycerol at a pH 8. The work-up involved acidification of the reaction mixture to pH 2, followed by extraction with EtOAc (3 × 30 mL). The organic phases were collected and washed with sat. $NaHCO_3$ (2 × 20 mL), brine (2 ×20 mL), dried over $MgSO_4$, filtered and the solvent evaporated under reduced pressure, affording the desired products as a yellow powder **7a** (41 mg, 70% isolated yield), a white powder **14a** (40 mg, 50% isolated yield), a white solid **16a** (12 mg, 15% isolated yield) and a white powder **17a** (42 mg, 51% isolated yield).

5. Analytical data

The following HPLC conditions were used: Column CNW Athena C18-WP, 5 µl, 100Å, eluents; A) H_2O, 0.1% formic acid, B) MeCN 0.1% formic acid.

Method I: 40% of B to 100% B in 5 min, 10 min at 100% B. 35 °C, flow 1 mL min^{-1}. **Method II:** 25% of B to 30% B in 5 min, 30% of B to 100% B in 1 min, 10 min at 100% B. 35 °C, flow 1 mL min^{-1}. Retention Times for acids and amide products are given in **Table S1**.

Table S1. Retention times for determination of acids and amides.

Entry	Compound	Method	Retention time (min)
1	**6**	I	7.1
2	**6a**	I	11.7
3	**6b**	I	10.5
4	**6c**	I	10.1
5	**6d**	I	10.6
6	**8**	I	4.5
7	**8a**	I	8.7
8	**8b**	I	8.5
9	**8d**	I	9.3
10	**9**	I	8.7
11	**9a**	I	11.9
12	**10**	I	3.9
13	**10a**	I	9.4
14	**10b**	I	8.9
15	**10d**	I	9.8
16	**11**	II	4.6
17	**11a**	II	12.7
18	**11b**	II	12.5
19	**11d**	II	12.9
20	**12**	I	3.8
21	**12a**	I	5.7
22	**13**	I	3.8
23	**13a**	I	6.2

24	**14**	I	3.9
25	**14a**	I	5.3
26	**15**	I	5.2
27	**15a**	I	6.1
28	**16**	I	4.6
29	**16a**	I	5.6
30	**17**	I	4.1
31	**17a**	I	5.2
32	**18**	I	4.1
33	**18a**	I	5.3
34	**19**	I	4.3
35	**19a**	I	5.4

Chiral HPLC analysis of Product 6d

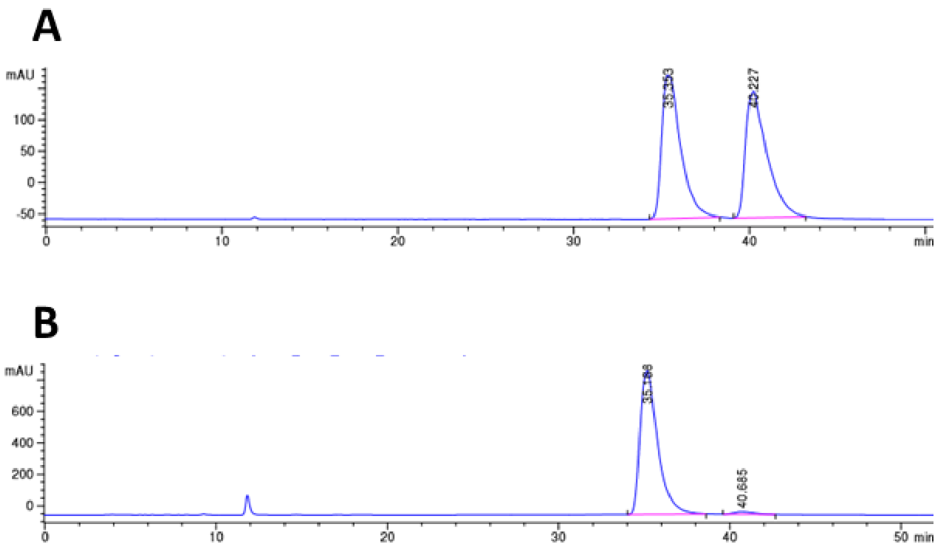

Figure S3. Chiral HPLC Analysis of product **6d** using a Chiralpak ID4 HPLC column using 20% isopropanol: 80% hexane as eluant with a flow rate of 1 mL min^{-1}. **A**: Racemic standard **6d**; Synthesis of a standard product of (*S*)-configuration from **6** and commercially sourced (*S*)-**d**, followed by chiral HPLC analysis confirmed the retention times of (*S*)- and (*R*)-**6d** as approximately 35.0 and 40.0 min respectively. **B**; product of reaction catalysed by McbA with 96% e.e.

6. Protein Crystallisation, Data Collection and Refinement

Mutagenesis

Mutation of lysine 483 to alanine was performed in an attempt to create an inactive McbA mutant K483A that might enable trapping of a stable reaction conformer. The mutation was introduced using site directed mutagenesis (SDM) with Pfu Turbo DNA polymerase (Agilent) with the wild-type McbA as a template. The following primers were used to introduce the desired mutation into the McbA gene: For *Rf*ACLR -Y137F Forward: (5' CGGCAGGTGCTCCGGATAAAGTTAAAGTTC 3') and Reverse: (5' TTATCCGGAGCACCTGCCGGTGTAACCGGA 3'). After SDM presence of the mutation was verified by sequencing.

Crystallization

The K483A mutant of McbA was expressed and purified as for the wild-type enzyme. A solution of the mutant at a concentration of 10 mg mL^{-1} which had been pre-incubated with 5 mM AMP and 10 mM β-carboline carboxylic acid **6** was subjected to crystallization screening using various commercial screens in sitting drop format using a Mosquito robot (TTP Labtech), in which 150 nL of protein solution is mixed with 150 nL of precipitant solution. Crystals were obtained in conditions containing 1.6 M sodium citrate pH 7.5. These crystals were washed in a cryoprotectant solution of 15% ethylene glycol in the mother the flash-cooled in liquid nitrogen prior to analysis at the Diamond Light Source.

Data collection, structure solution and refinement

Data were collected on beamline i03 at the Diamond Light Source and were processed and integrated using XDS[19] and scaled using SCALA[20]within the Xia2[21] processing system. Data collection statistics can be found in **Table S2.** The structure was solved with MOLREP[22] using the structure of protein acetyltransferase RpPAT from *Rhodopseudomonas palustris* (28% sequence identity, PDB code 4GXQ)[23] as the molecular replacement model. The structures were refined using iterative cycles of the programmes COOT[24] and REFMAC5.[25] After building of the protein backbone, side chains and water molecules, residual density was present in the omit map all five active sites. This could be modelled in four subunits, A-D, each in the 'adenylation' conformation as AMP and β-carboline acid **6**. In subunit E, in the 'amidation' conformation, density for **6** was again clearly visible but the AMP peak lacked density for the phosphate group. Coordinate and library files for **6** were prepared using ACEDRG.[26] Refinement statistics for all structures can be found in **Table S1**. Coordinates and structure factor files have been deposited in the Protein Data Bank (PDB) with the accession code 6H1B.

Table S2. Data Collection and Refinement Statistics for McbA K483A in complex with **6** and AMP. Numbers in brackets refer to data for highest resolution shells.

	McbA K483A in complex with **6** and AMP
Beamline	Diamond I03
Wavelength (Å)	0.97624
Resolution (Å)	108.83-2.80 (2.86-2.80)
Space Group	$P2_12_12_1$
Unit cell (Å)	a = 118.23; b = 130.74; c = 196.38
	$\alpha = \beta = \gamma = 90°$
No. of molecules in the asymmetric unit	5
Unique reflections	75674 (4421)
Completeness (%)	100.0 (100.0)
R_{merge} (%)	0.26 (1.13)
$R_{\mathrm{p.i.m.}}$	0.11 (0.46)
Multiplicity	13.2 (13.4)
$\langle I/\sigma(I) \rangle$	8.3 (2.6)
Overall *B* factor from Wilson plot (Å^2)	41
$CC_{1/2}$	0.99 (0.67)
R_{cryst}/R_{free} (%)	20.5/24.5
r.m.s.d 1-2 bonds (Å)	0.010
r.m.s.d 1-3 angles (^{o})	1.41
Avge main chain B (Å^2)	52
Avge side chain B (Å^2)	54
Avge water B (Å^2)	29
Avge ligand B for AMP (Å^2)	54
Avge ligand B for **6** (Å^2)	45

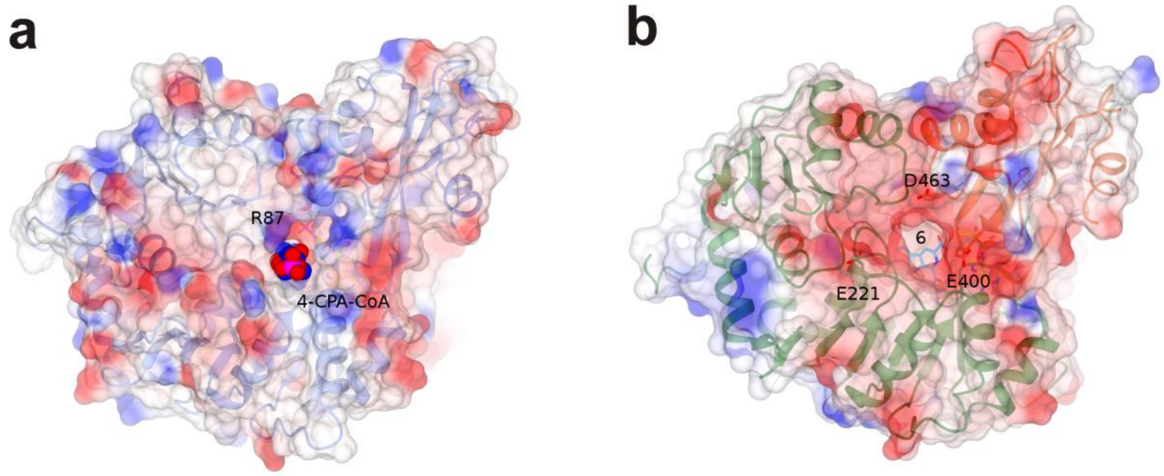

Figure S3. **a**: Structure of 4-chlorobenzoyl-CoA ligase (4CBL, 3CW9)[27] in the thiolation conformation, in complex with 4-chlorophenacyl-CoA (4-CPA-CoA), showing the electrostatic surface of the protein and the access channel for the phosphopantotheinate (displayed in sphere format) in the thiolation reaction. The adenosine of CoA has been removed in this Figure for clarity. **b**: Structure of McbA in the amidation conformation, showing the electrostatic surface and putative access channel for an amine substrate in an amidation reaction. Carboxylic acid substrate **6** can be seen through the relevant channel on the enzyme surface.

7. References

[1] Still, W. C.; Kahn, M.; Mitra, A. *J. Org. Chem.* **1978**, *43*, 2923.

[2] Atkin, K.E.; Reiss, R.; Turner, N.J.; Brzozowski, A.M.; Grogan, G. *Acta Crystallogr. Sect. F. Struct. Biol. Cryst. Commun.,* **2008**, 64, 182-185.

[3] Battini, N.A.; Padala, K.; Mupparapu, N.; Vishwakarma, R.A.; Ahmed, Q.N. *RSC Adv.*, **2014**, 4, 26258–26263.

[4] Nemet, I.; Varga-Defterdarovic ́ L. *Bioorg. Med. Chem*., **2008**, 16, 4551–4562.

[5] Lin, G.; Wang, Y.; Zhou, Q.; Wang, J.; Yang, T.; Wang, Z.; Lu, T. *Synth. Commun*., **2012**, 42, 1895-1910.

[6] Lingam, Y.; Rao, D. M.; Rao, J. S.; Venu, B. K.; Mohammad, A. K.; Kumar, G. S.; Islam, A. *Org. Chem. Ind. J.,* **2008**, 4, 28-31.

[7] Shinji, T.; Tominari, C.; Asuka, O.; Takashi, N.; Shiroh, W.; Noriyuki, H.; Satoshi, H. *Heterocycles*, **2013**, 87, 357-367.

[8] Yang, M.L.; Kuo, P.C.; Damu, A.G.; Chang, R.J.; Chiouc, W.F.; Wua, T.S. *Tetrahedron*, **2006**, 62, 10900–10906.

[9] Bohang, S.; Toshio, M.; Hisashi, M.; Supinya, T.; Jun, W.L.; Shoichi, H.; Masayuki, Y., *J. Nat. Prod.* **2004**, 67, 1464-1469.

[10] Kornsalkulkarn, J.; Saepua, S.; Boonruangprapa, T.; Suphothina, S.; Thongpanchang, C.; *Phytochem. Lett.* **2013**, 6, 491-494,

[11] Yin, W.; Majumder, S.; Clayton, T.; Petrou, S.; Van Linn, M.L.; Namjoshi, O.A.; Ma, C.; Cromer, B.A.; Roth, B.L.; Platt, D.M.; Cook, J.M. *Bioorg. Med. Chem*., **2010**, 18, 7548–7564.

[12] Dunetz, J.R.; Xiang, Y.; Baldwin, A.; Ringling, J. *Org.Lett.,* **2011**, 13, 5048-5051.

[13] Huang, H.; Yao, Y.; He, Z.; Yang, T.; Ma, J.; Tian, X.; Li, Y.; Huang, C.; Chen, X.; Li, W. *J. Nat. Prod.* **2011**, 74, 2122-2127.

[14] Funder, E. D.; Trads, J. B.; Gothelf, K. V.; *Org. Biomol. Chem.* **2015**, 13, 185–198.

[15] Carlock, J. T.; Bradshaw, J. S.; Stanovnik, B.; Tišler, M.; *J. Org. Chem.* **1977**, 42, 1883–1885.

[16] Lawrence, R. P. N. J.; Sebti, S. M.; US Pat. 9 616 064, 2012.

[17] Noda, H.; Bode, J. W.; *Chem. Sci.* **2014**, 5, 4328–4332.

[18] Prasad, V.; Kale, R. R.; Mishra, B. B.; Kumar, D.; Tiwar, V. K.; *Org. Lett.*, **2012**, 14, 2936 – 2939.

[19] Kabsch, W. *Acta Crystallogr. Sect. D. Biol. Crystallogr.* **2010**, *66*, 125-132.

[20] Evans, P. *Acta Crystallogr. Sect. D. Biol. Crystallogr.* **2006**, *62*, 72-82.

[21] Winter, G. *J. Appl. Cryst*., **2010**, *3*, 186-190.

[22] Vagin, A.; Teplyakov, A. *J. Appl. Crystallogr.* **1997**, *30*, 1022-1025.

[23] Crosby, H.A.; Rank, K.C.; Rayment, I.; Escalante-Semerena, J.C. *J. Biol. Chem.* **2012**, *287*, 41392-41404.

[24] Emsley, P.; Cowtan, K. *Acta Crystallogr., Sect D: Biol. Crystallogr.* **2004**, *60*, 2126-2132.

[25] Murshudov, G.N.; Vagin, A.A.; Dodson, E.J. *Acta. Crystallogr. Sect. D. Biol. Crystallogr.* **1997**, *53*, 240-255.

[26] F. Long, R. A. Nicholls, P. Emsley, S. Grazulis, A. Merkys, A. Vaitkus, G. N. Murshudov, *Acta Crystallographica Section D* **2017**, *73*, 112-122.

[27] R. Wu, J. Cao, X. Lu, A. S. Reger, A. M. Gulick, D. Dunaway-Mariano, *Biochemistry* **2008**, *47*, 8026-8039.

8. Compound NMR spectra

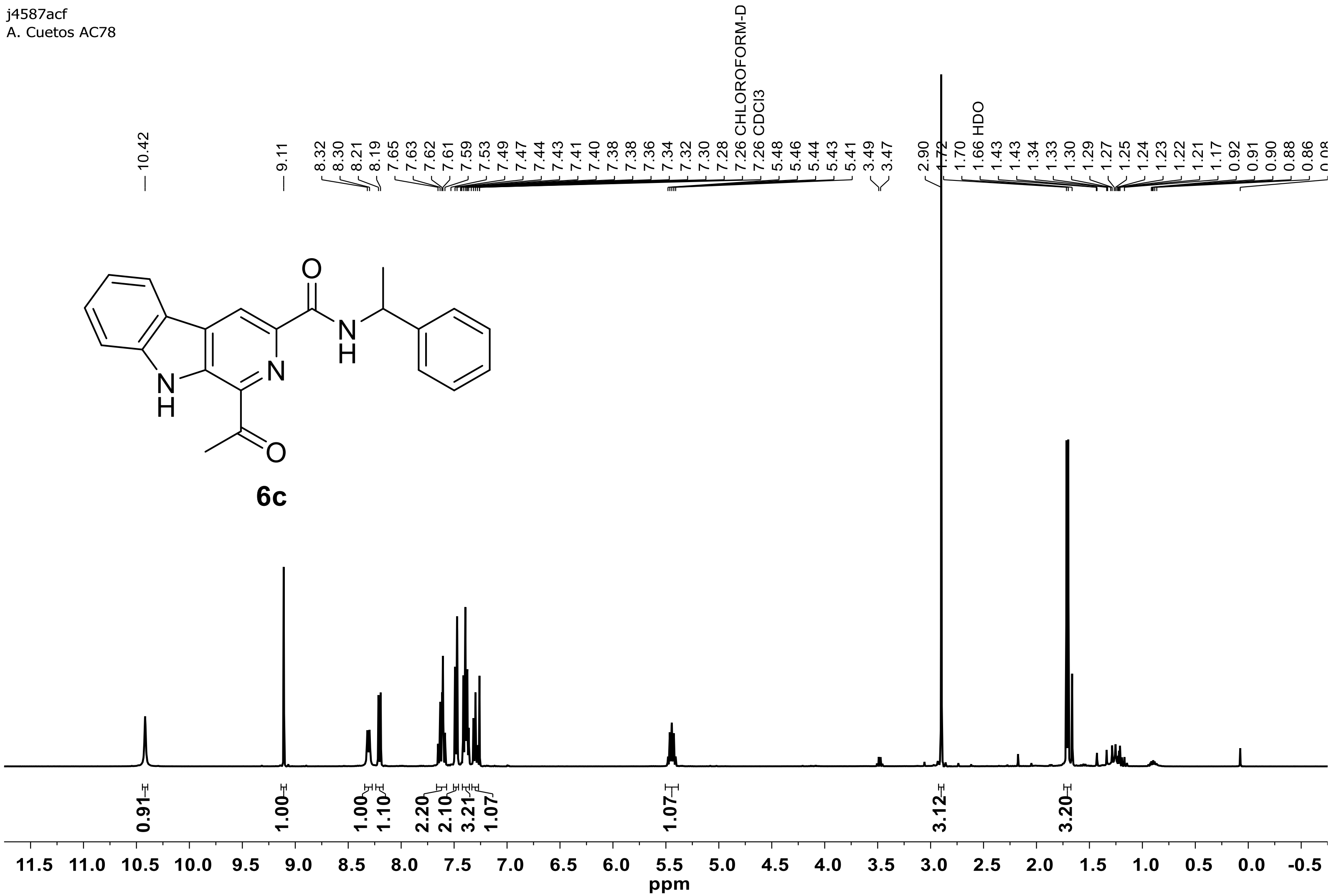

^{1}H NMR spectrum of compound 6c ($CDCl_3$; the signal at δ2.90 is shown off-scale).

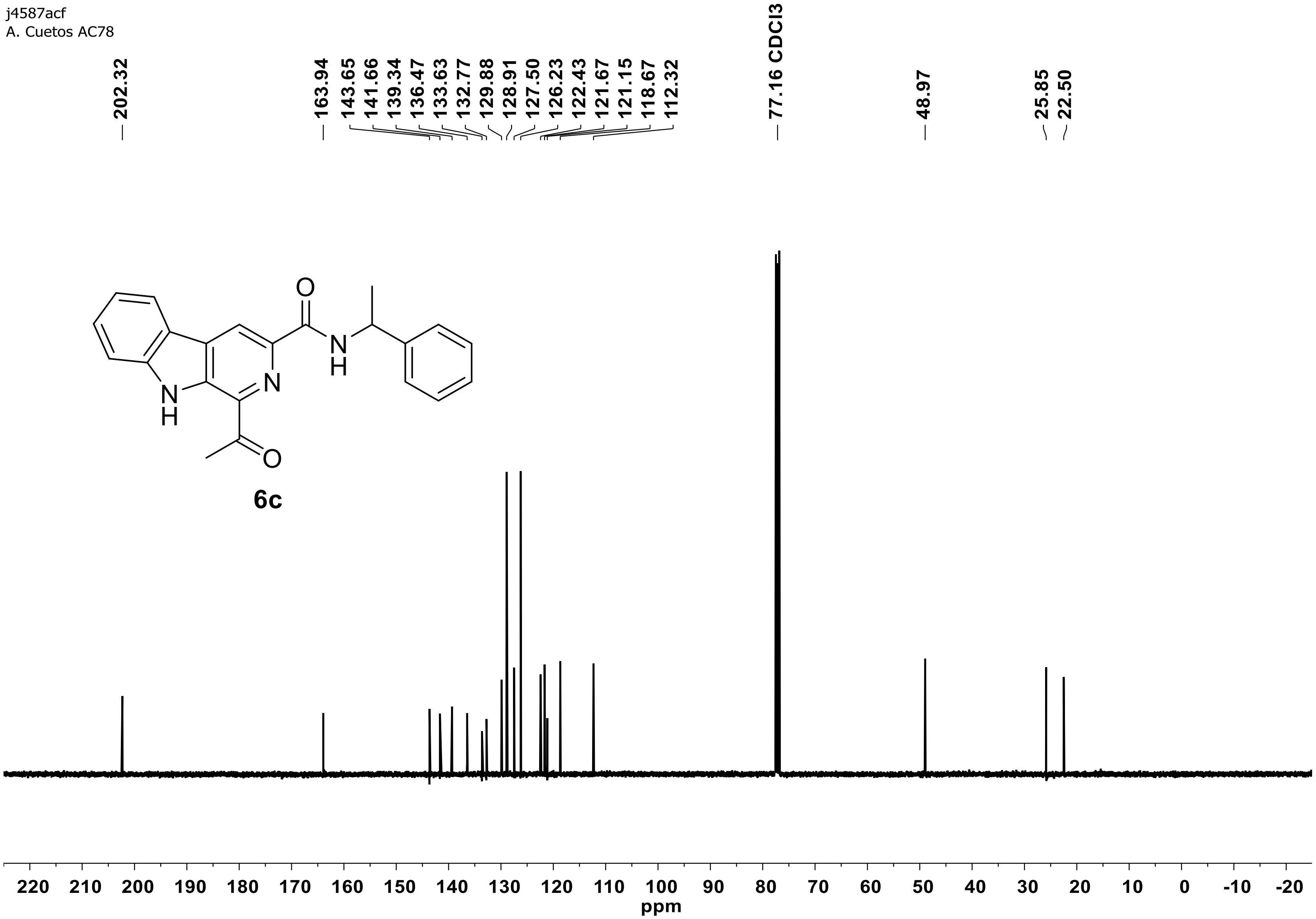

^{13}C NMR spectrum of compound 6c ($CDCl_3$).

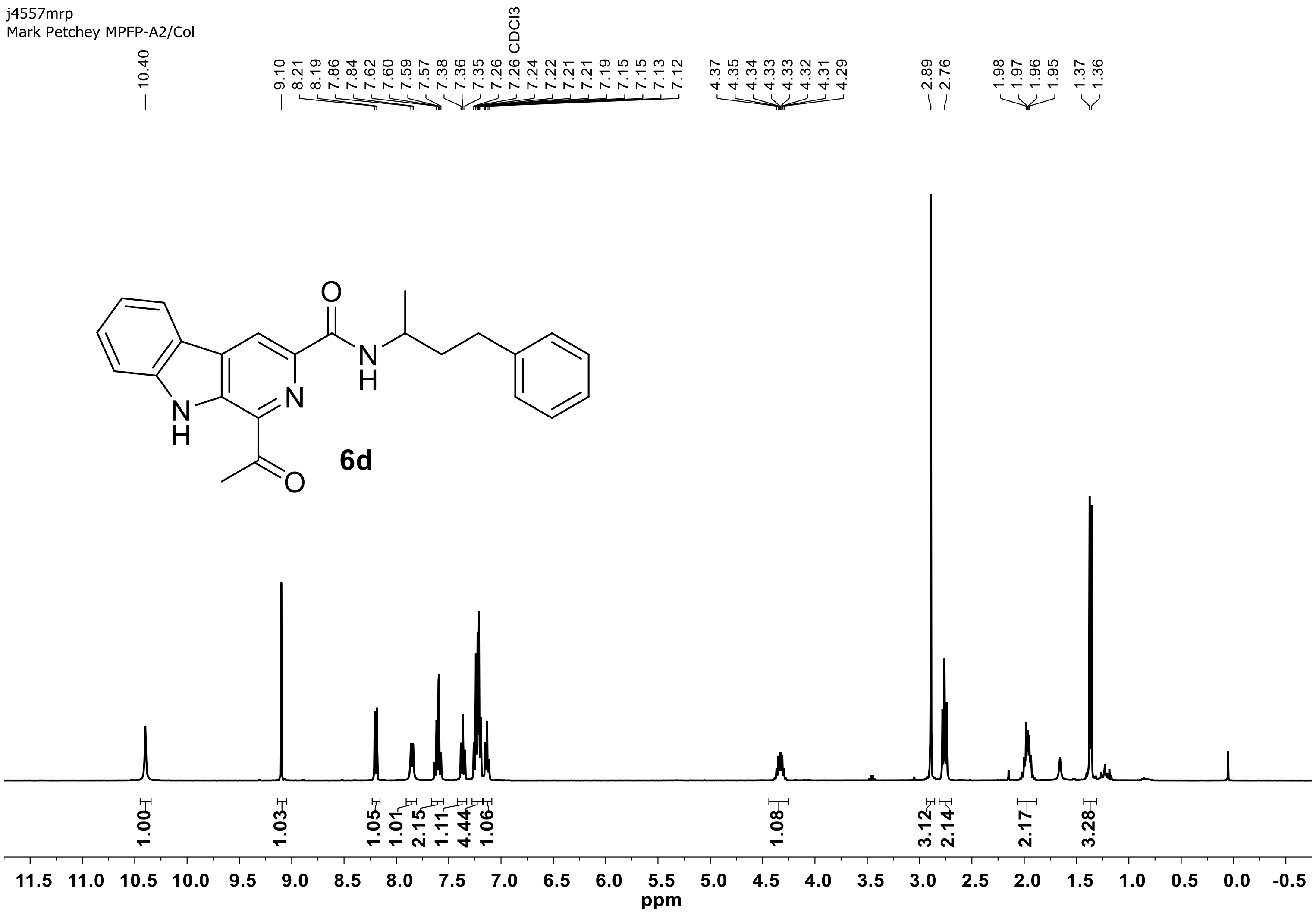

^{1}H NMR spectrum of compound 6d ($CDCl_3$).

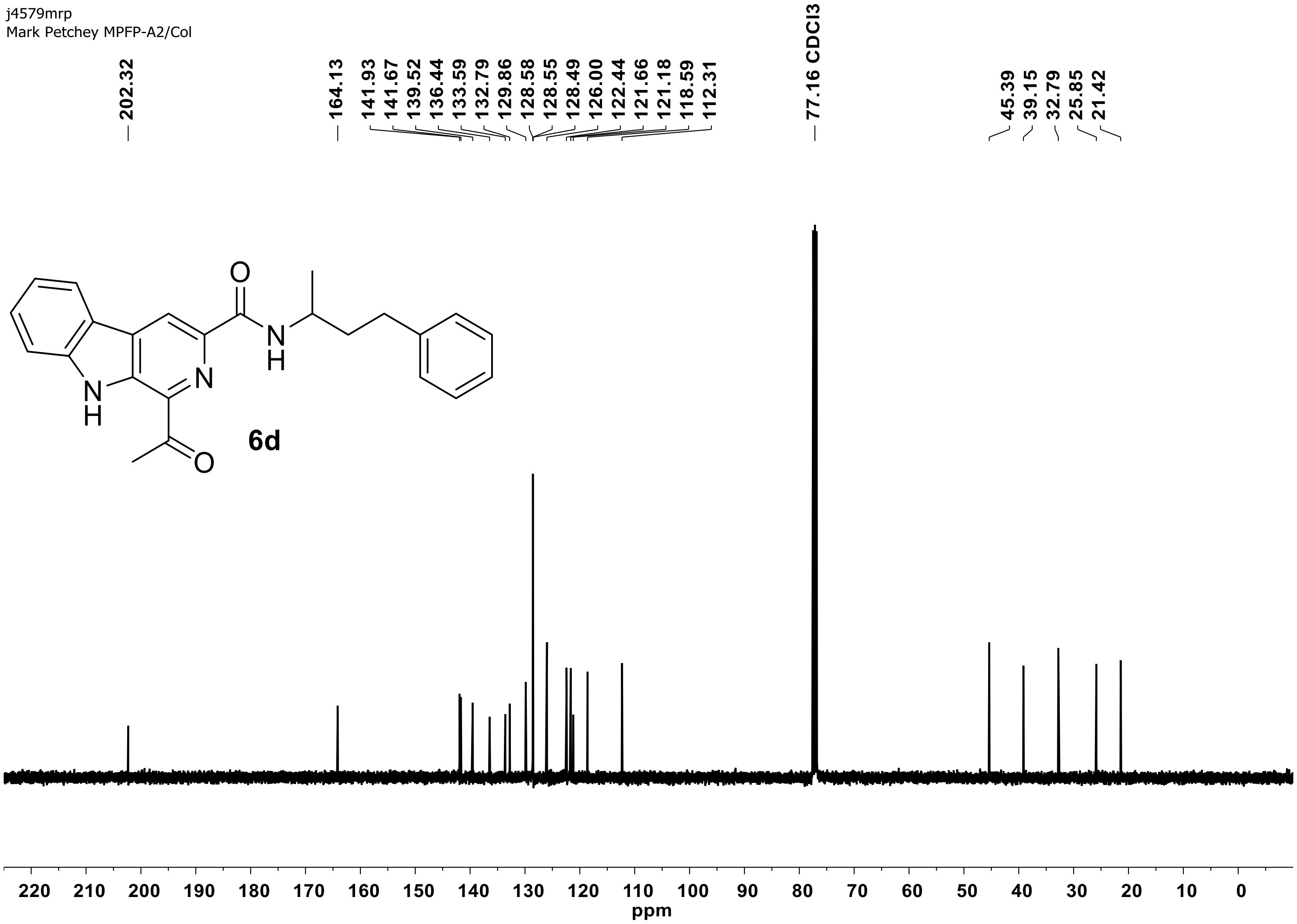

^{13}C NMR spectrum of compound 6d ($CDCl_3$).

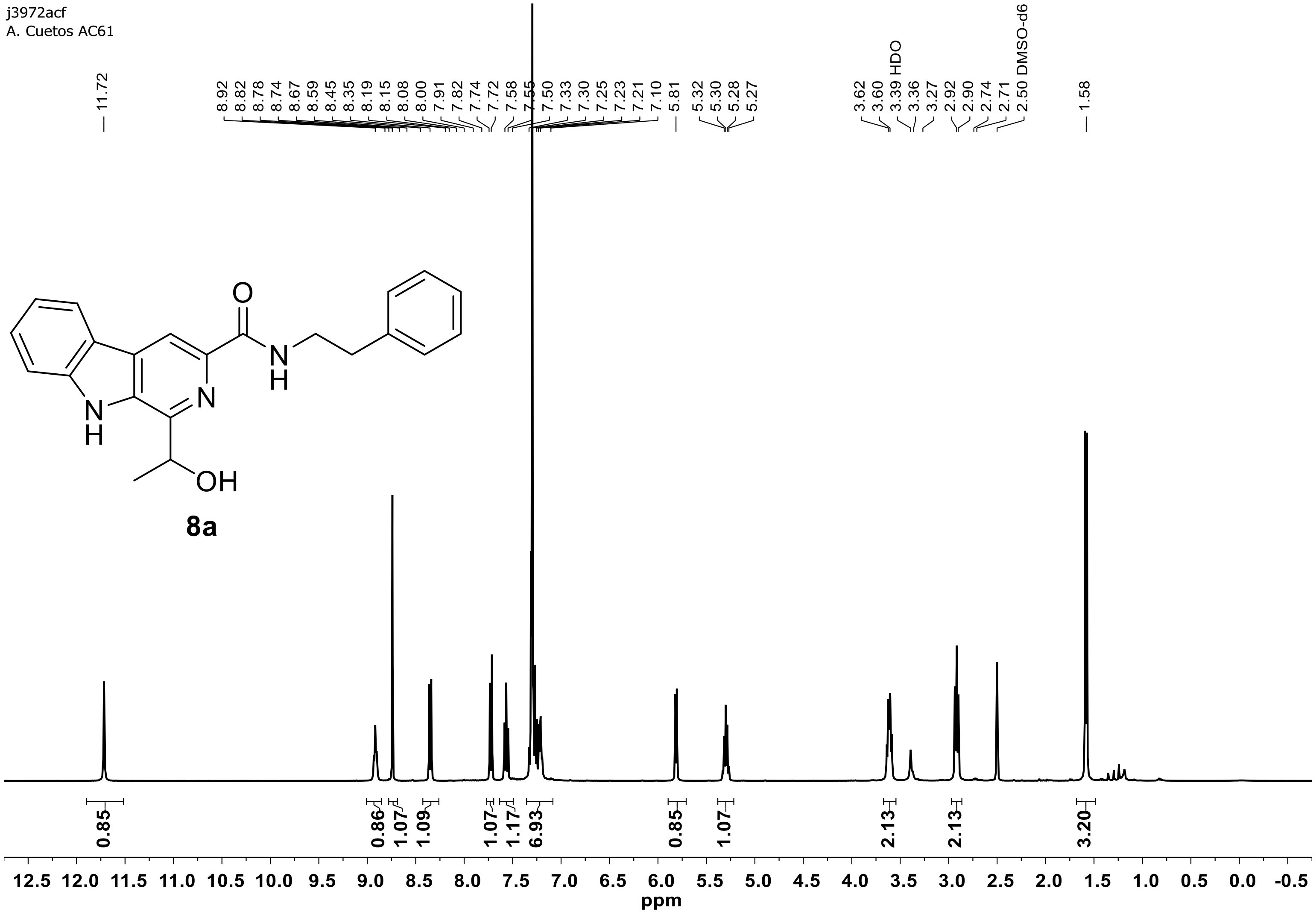

^{1}H NMR spectrum of compound 8a (DMSO-d_6; residual $CHCl_3$ is shown off-scale).

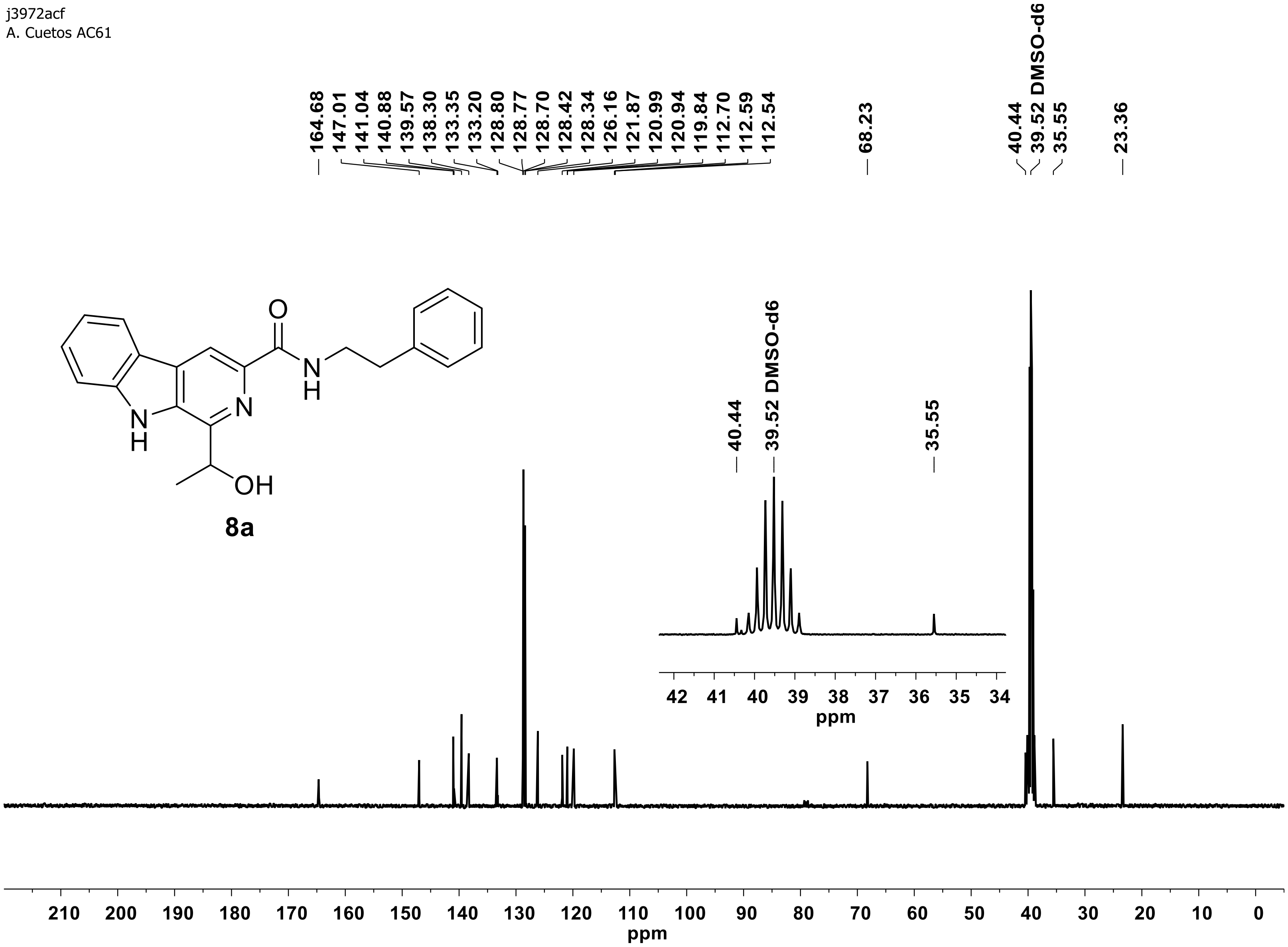

^{13}C NMR spectrum of compound 8a (DMSO-d_6).

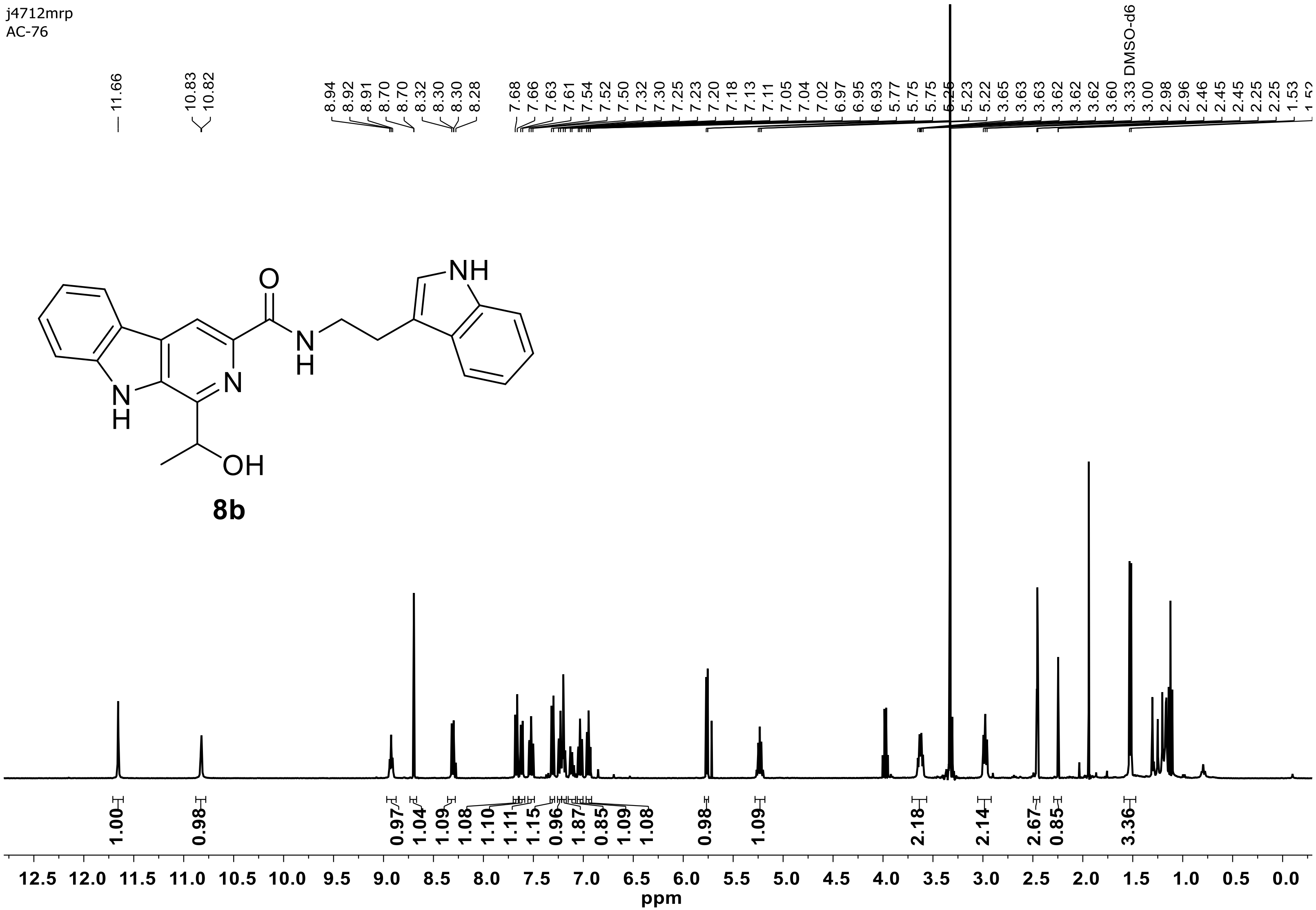

^{1}H NMR spectrum of compound 8b (DMSO-d_6). Trace EtOAc present at δ 1.12 (t), 1.94 (s), and 3.98 (q)

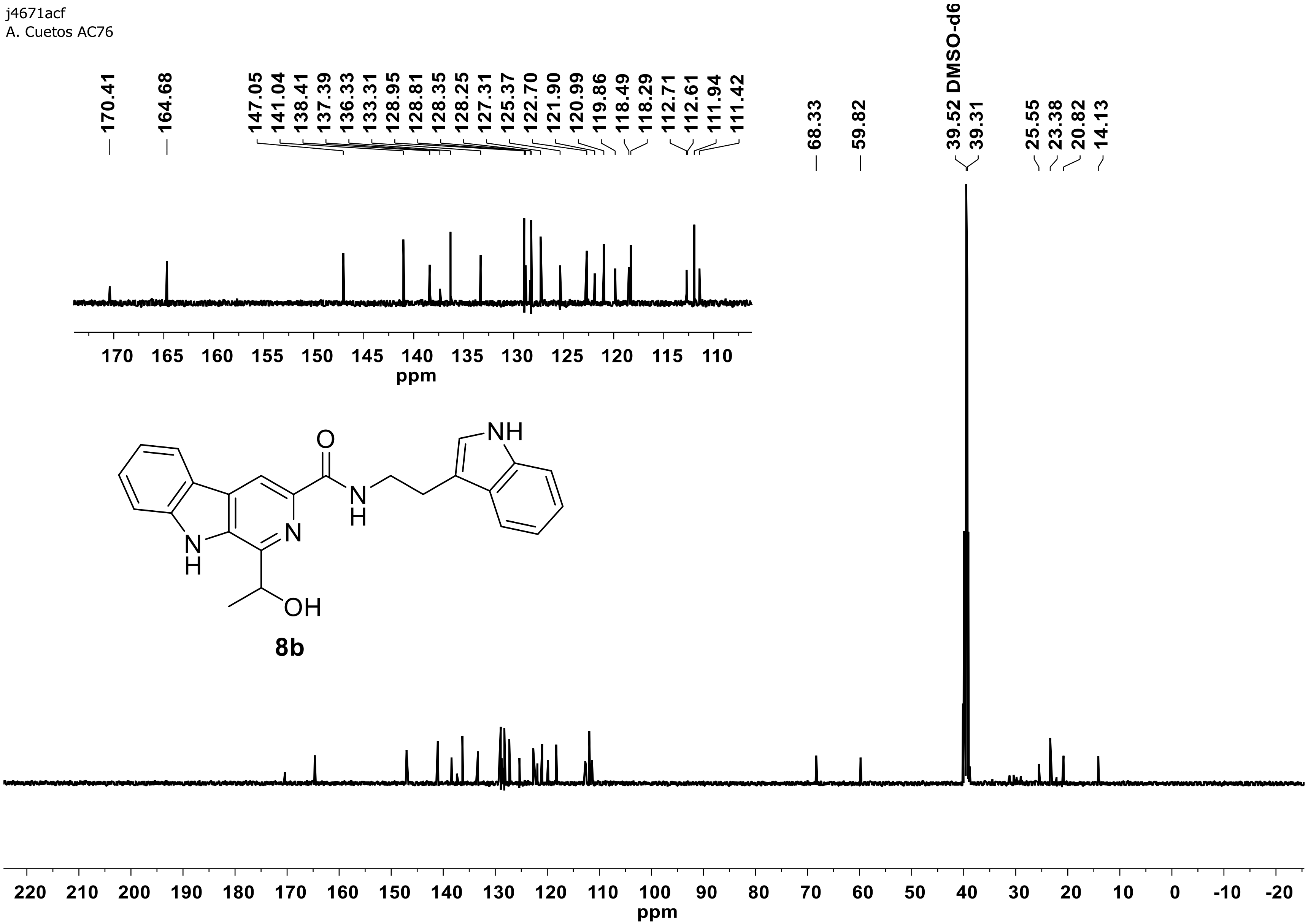

^{13}C NMR spectrum of compound 8b (DMSO-d_6).

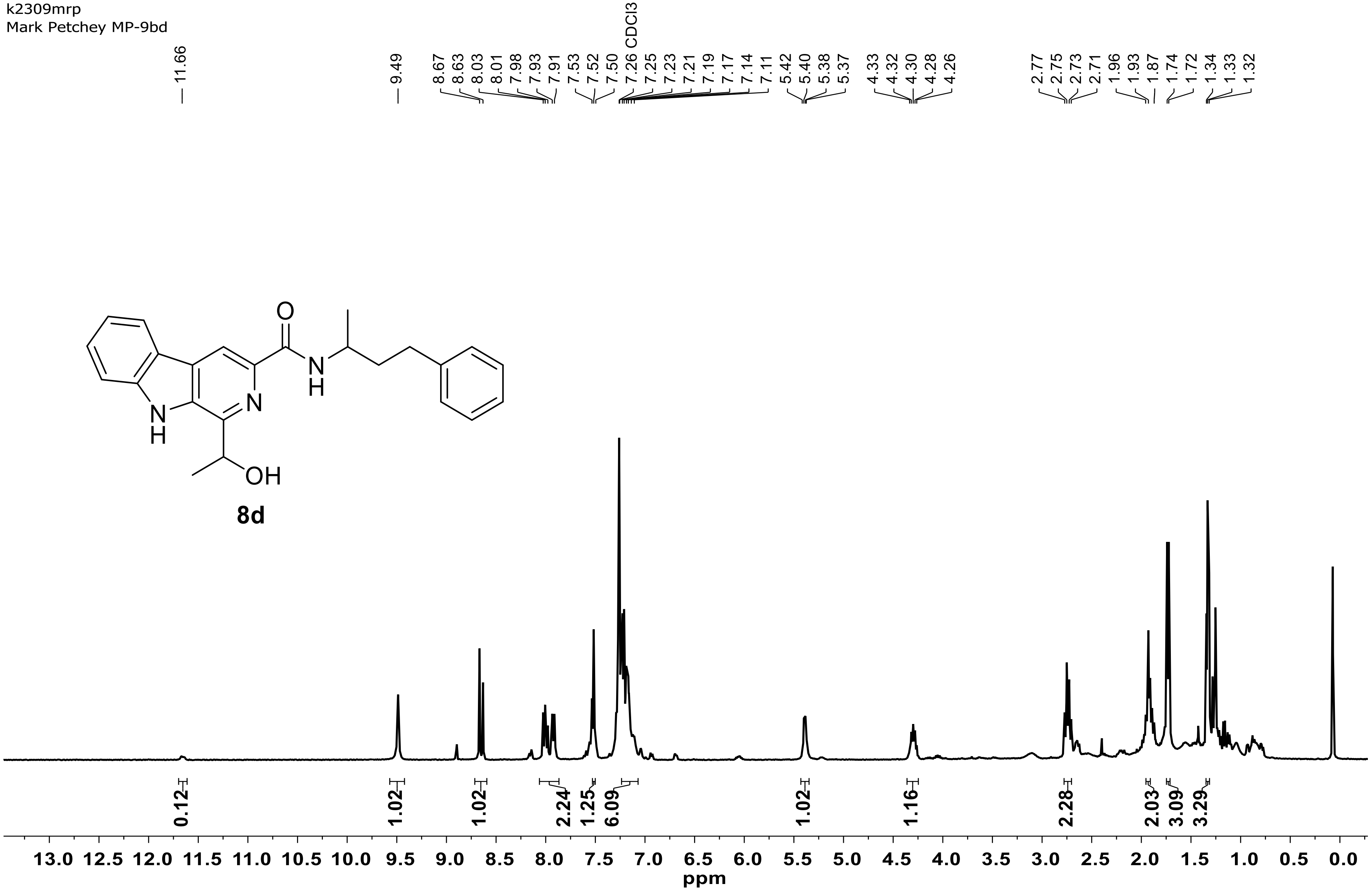

8d

^{1}H NMR spectrum of compound 8d ($CDCl_3$).

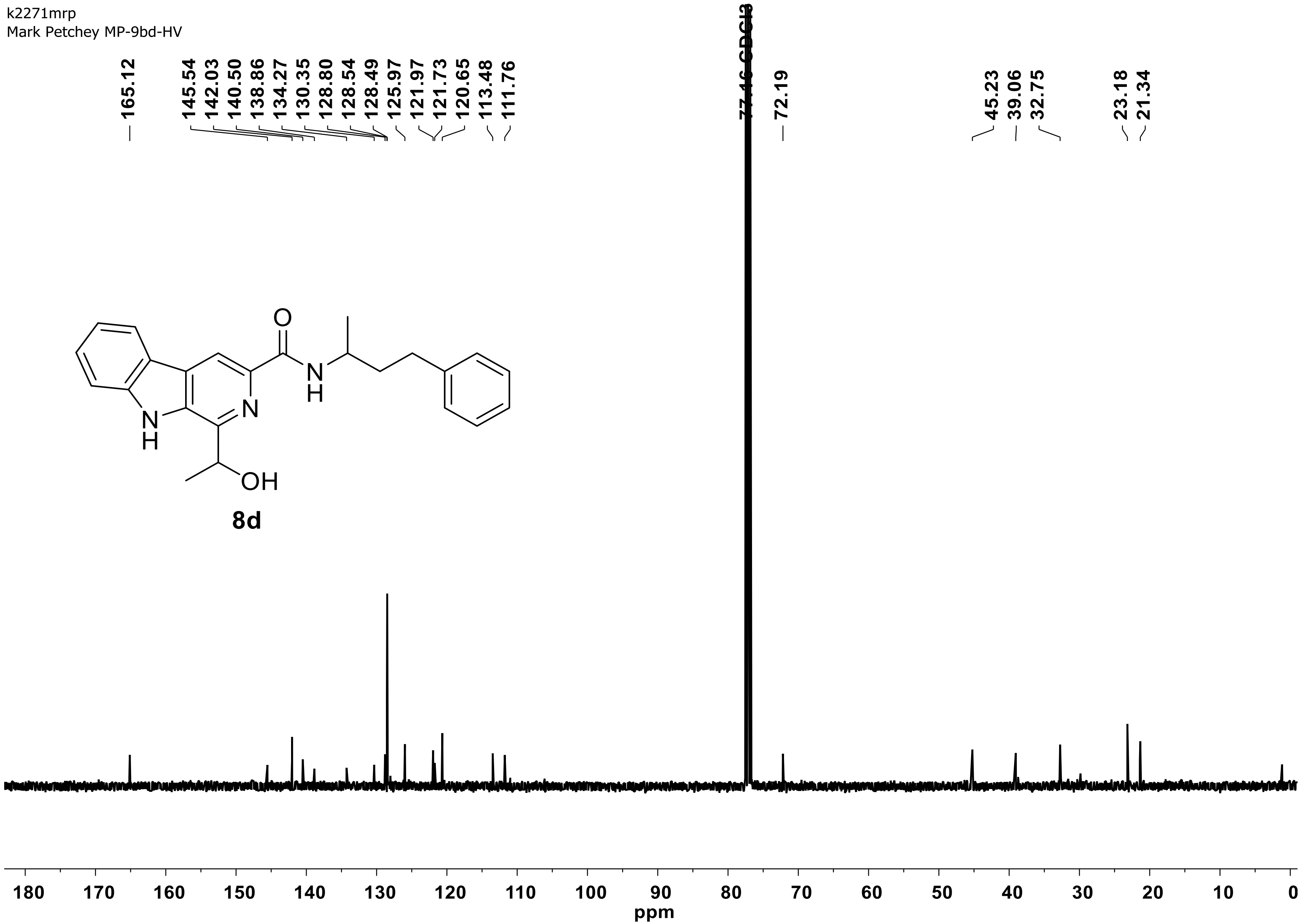

^{13}C NMR spectrum of compound 8d ($CDCl_3$).

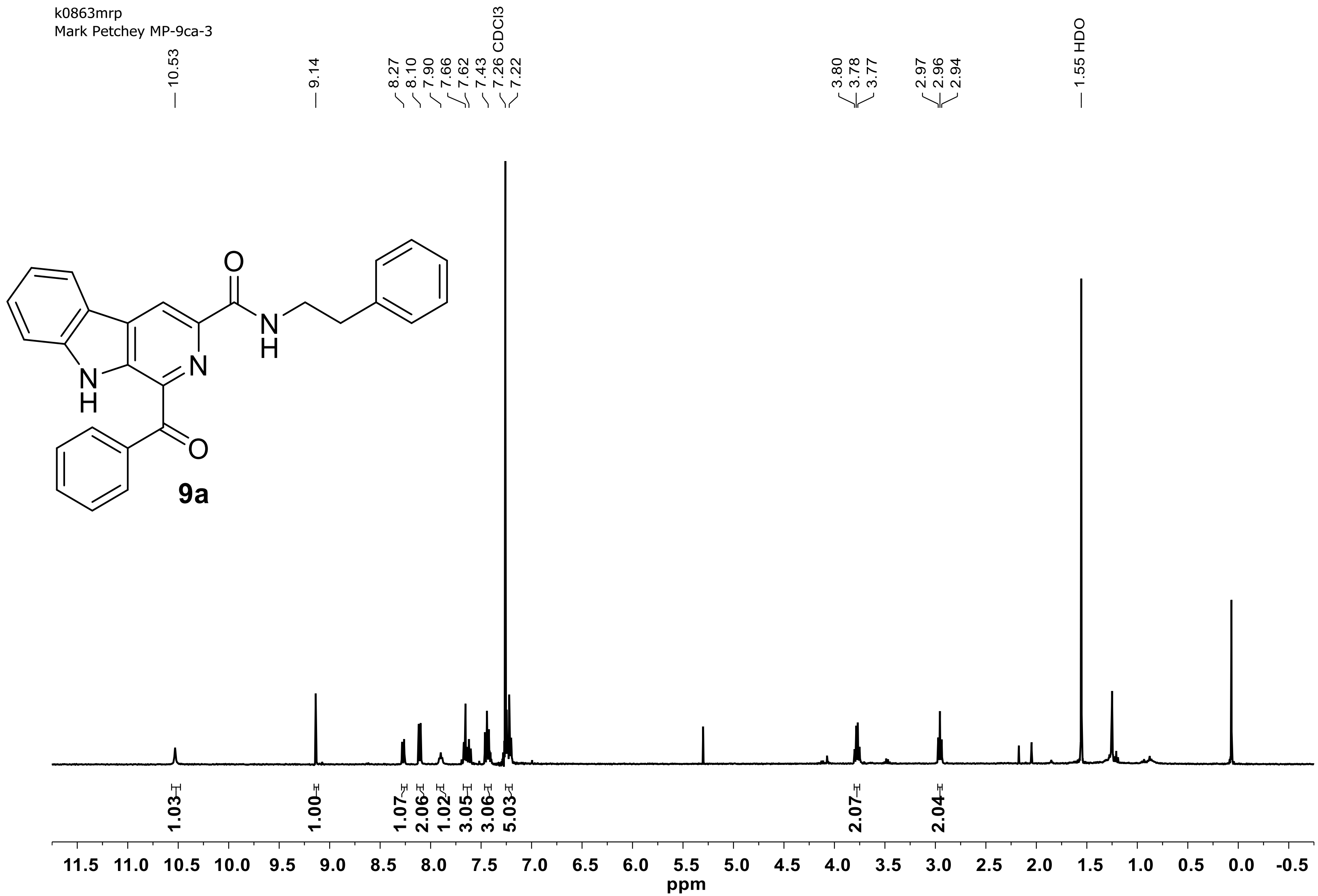

^{1}H NMR spectrum of compound 9a ($CDCl_3$).

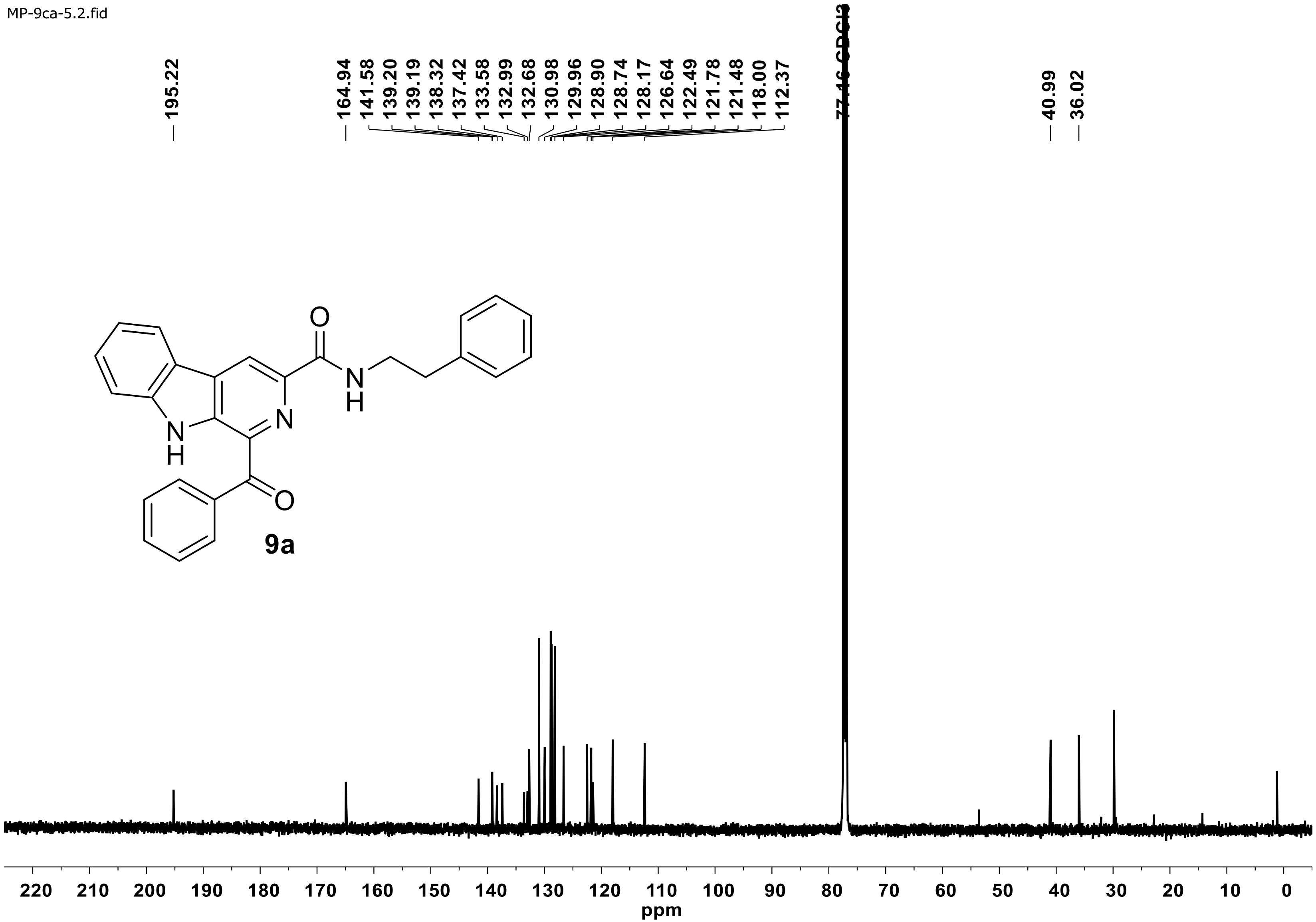

^{13}C NMR spectrum of compound 9a ($CDCl_3$).

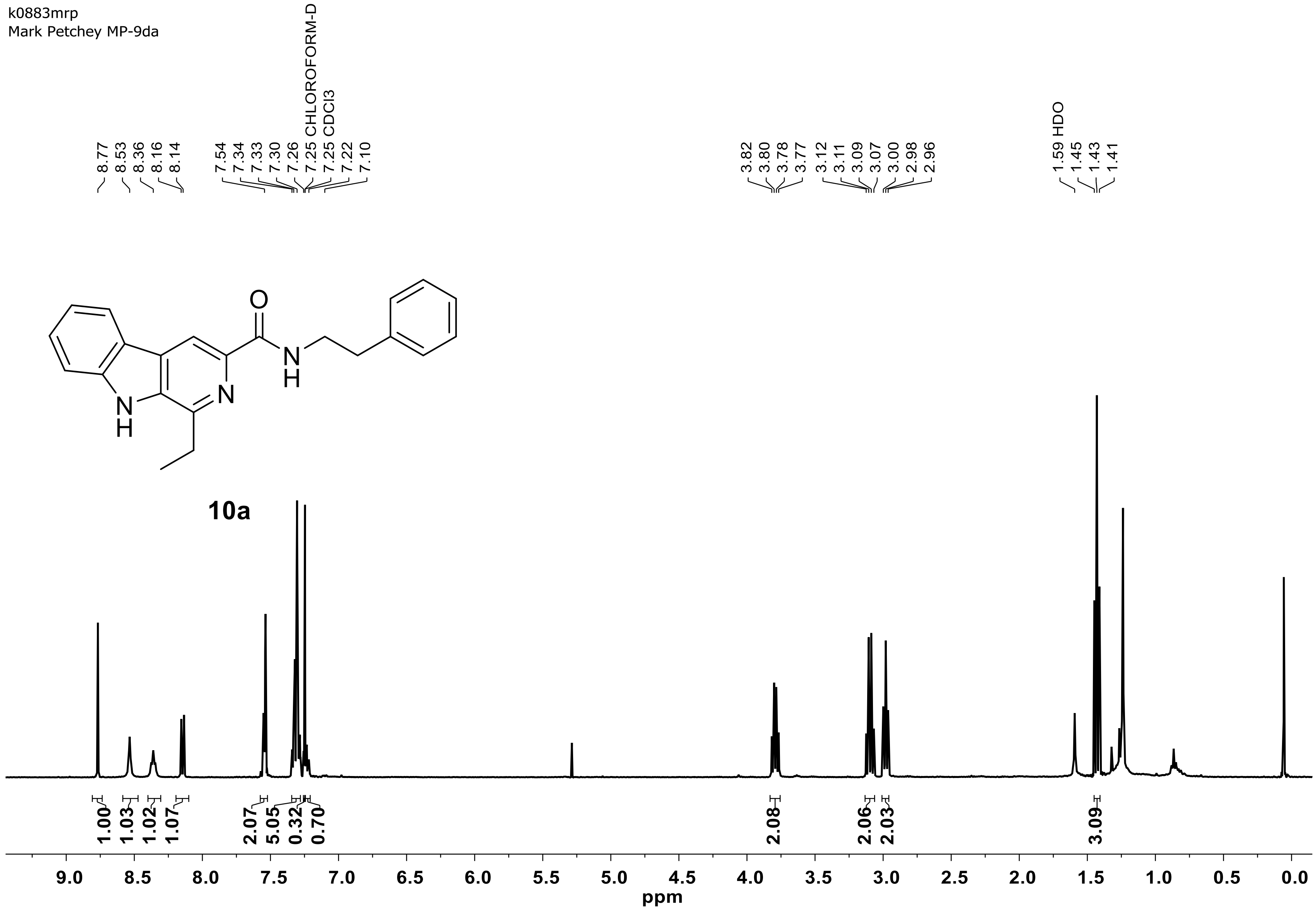

^{1}H NMR spectrum of compound 10a ($CDCl_3$; referenced to residual CH_2Cl_2 at δ5.31).

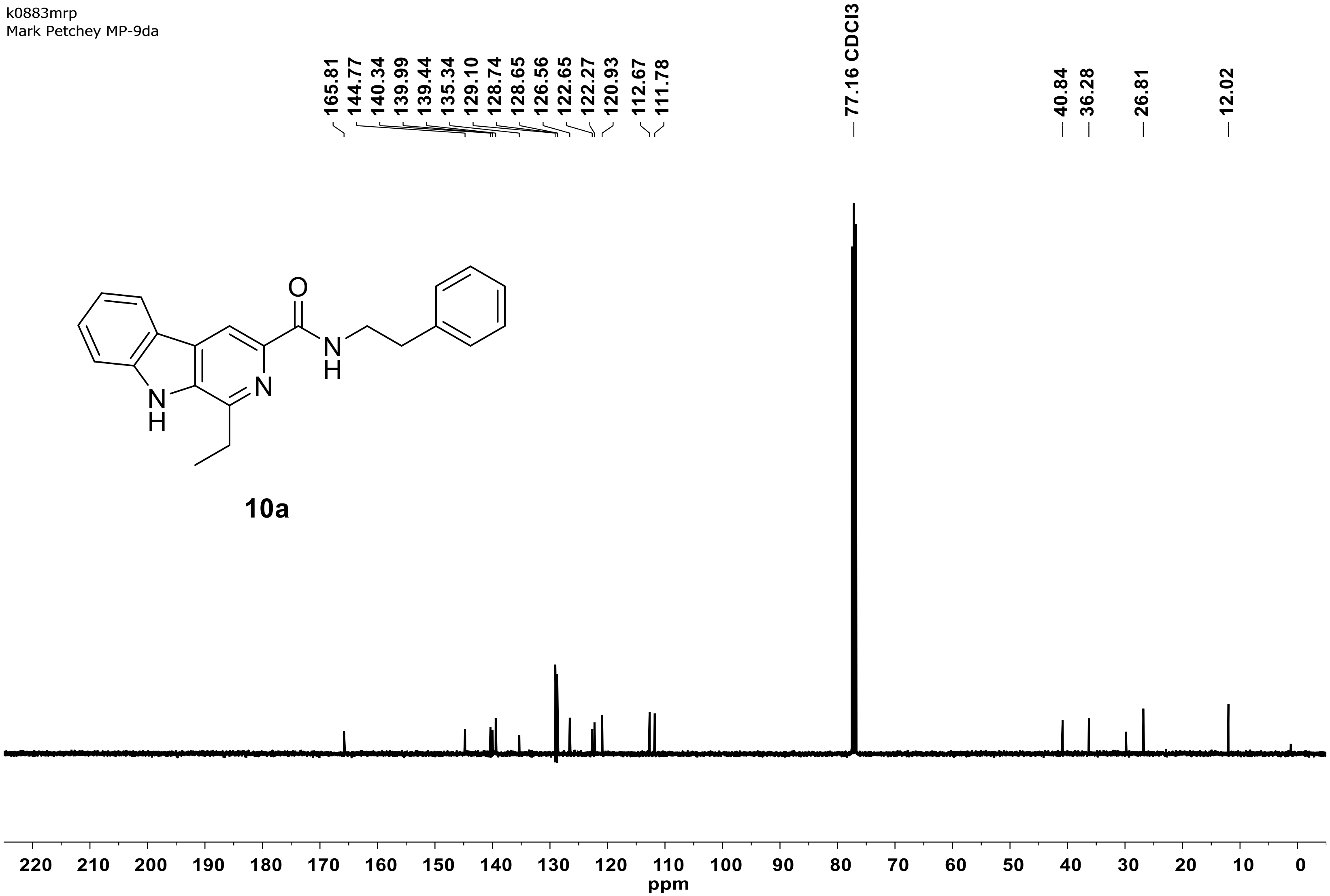

^{13}C NMR spectrum of compound 10a ($CDCl_3$).

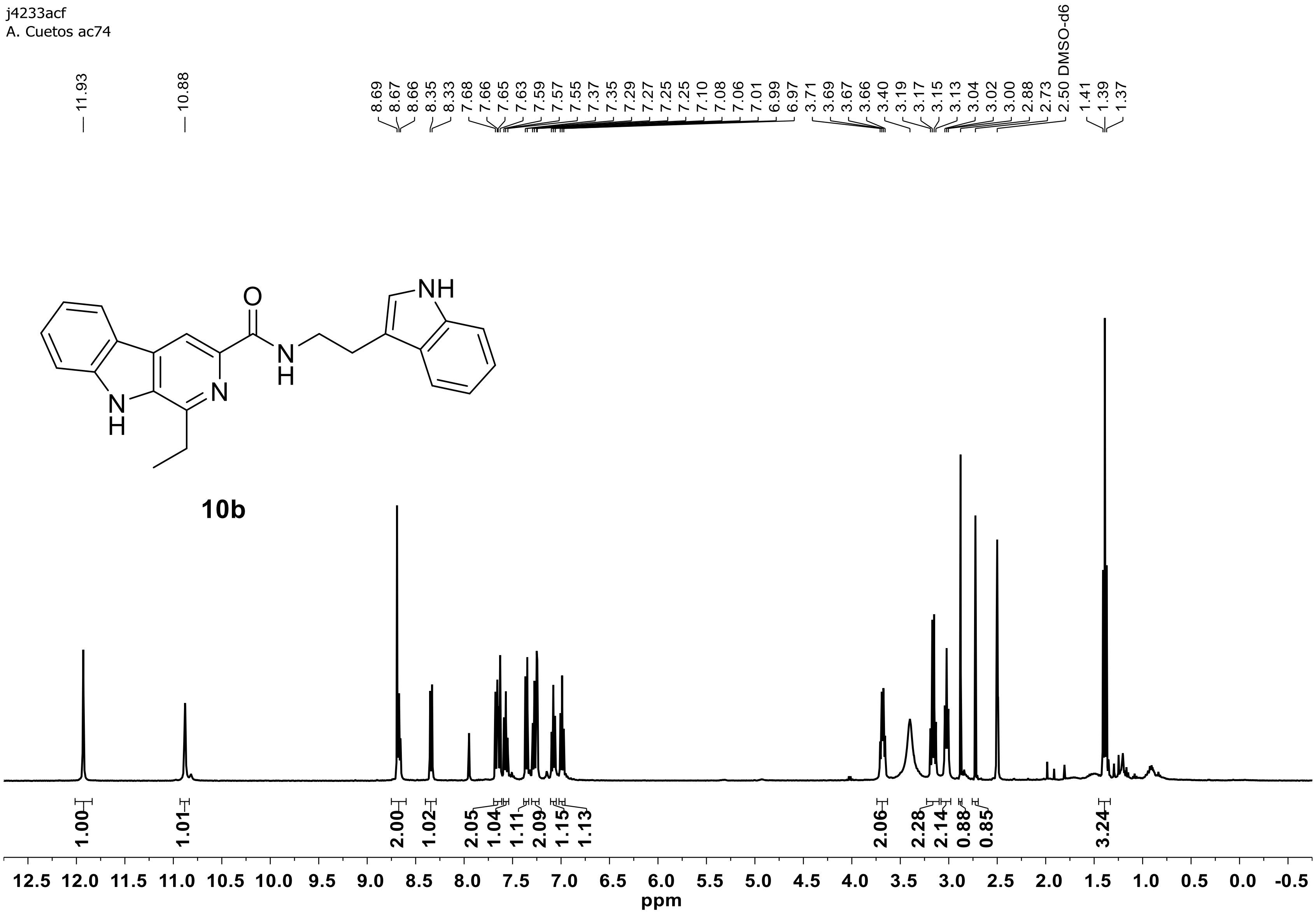

^{1}H NMR spectrum of compound 10b (DMSO-d_6).

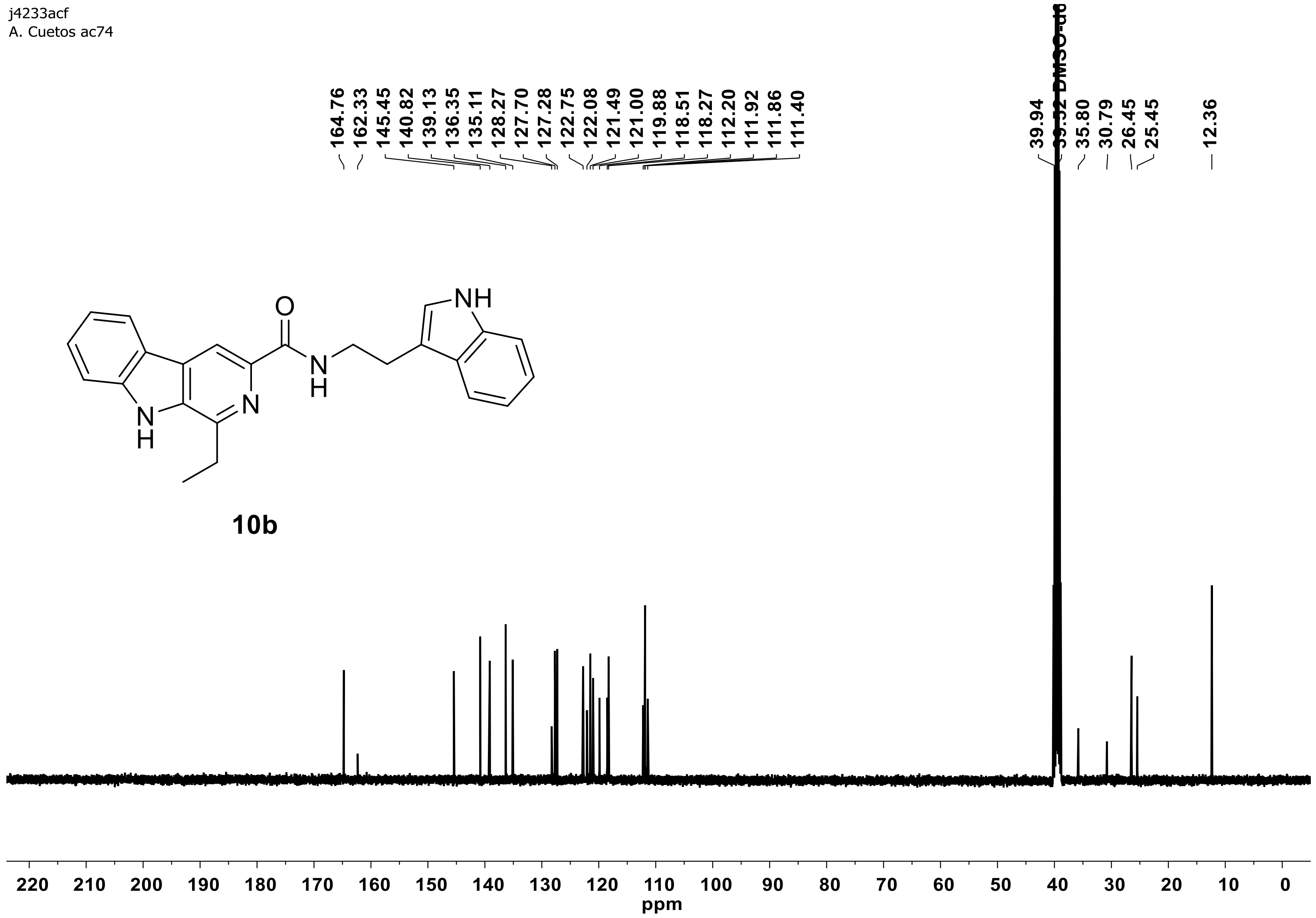

^{13}C NMR spectrum of compound 10b (DMSO-d_6; carbon signal for DMSO-d_6 is shown off-scale).

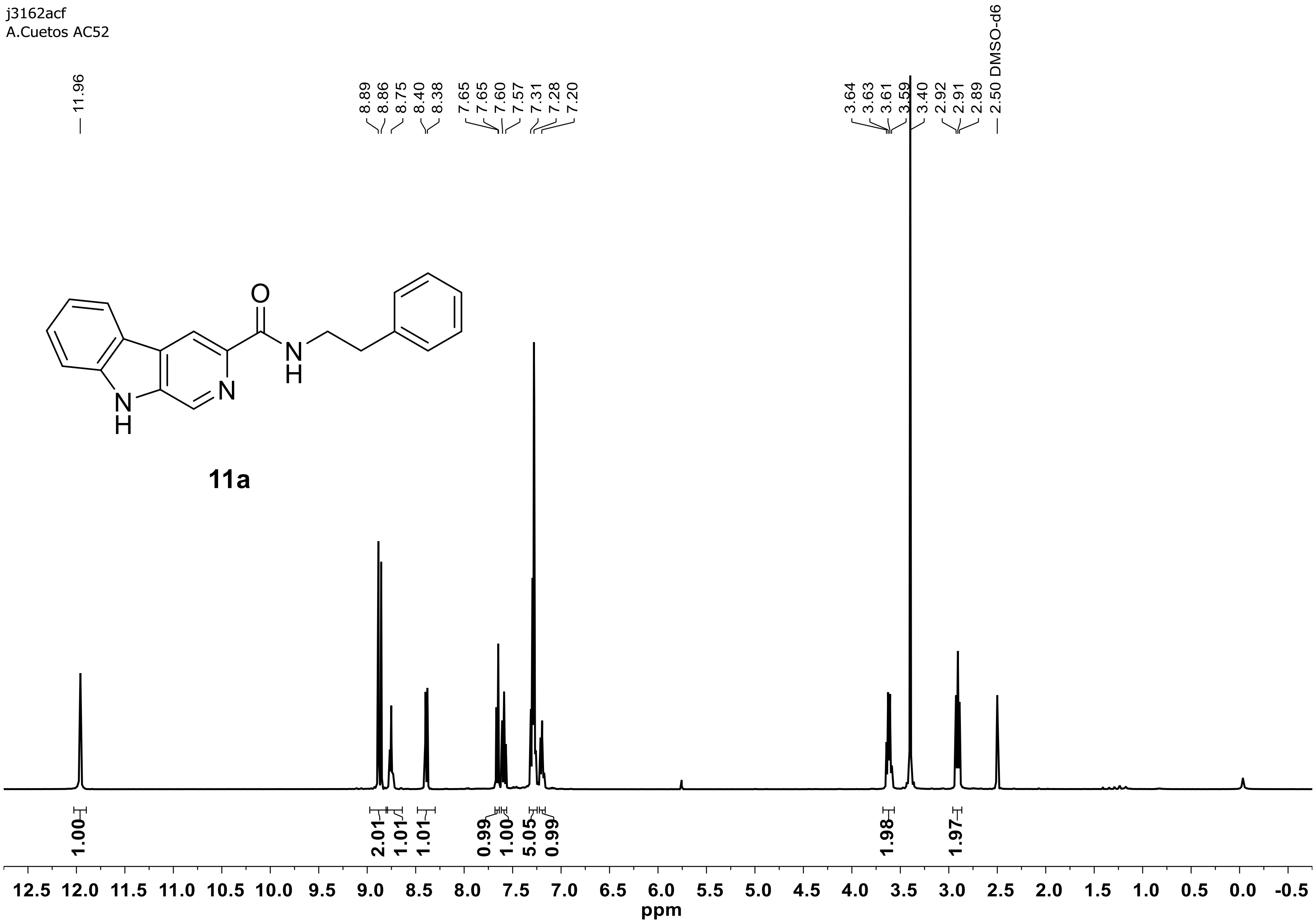

^{1}H NMR spectrum of compound 11a (DMSO-d_6; residual H_2O at δ3.40 is shown off-scale).

j3162acf
A.Cuetos AC52

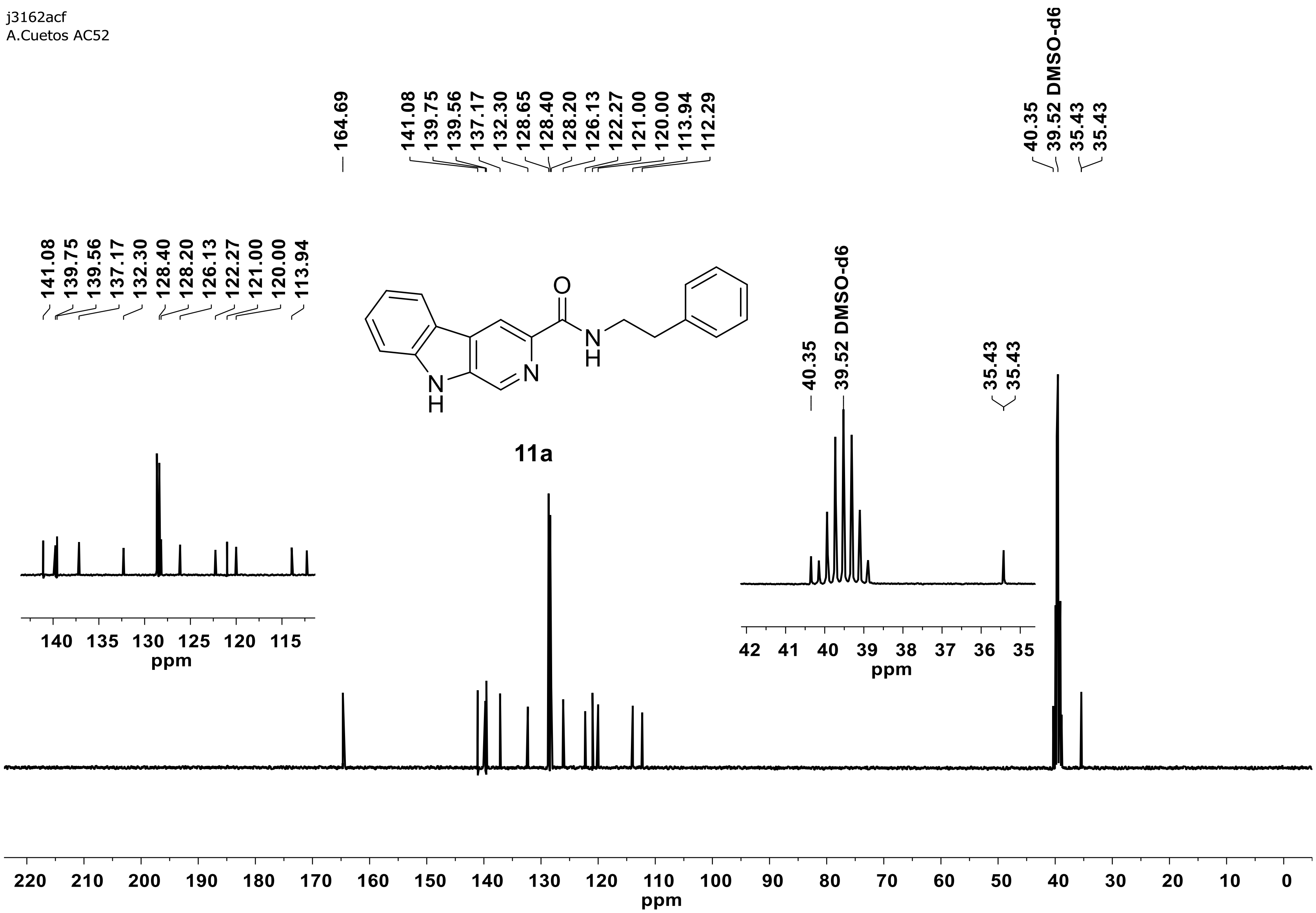

^{13}C NMR spectrum of compound 11a (DMSO-d_6).

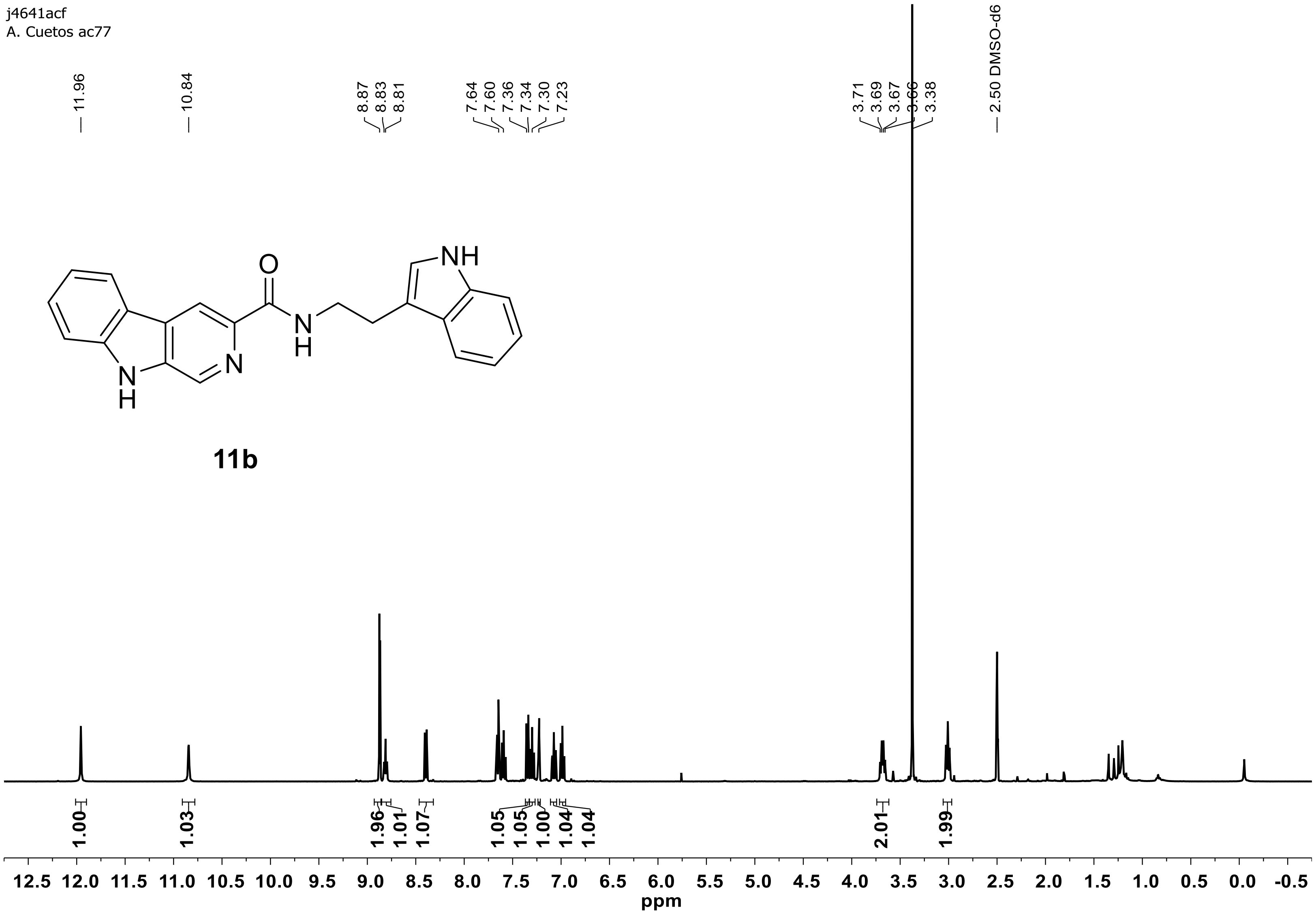

^{1}H NMR spectrum of compound 11b (DMSO-d_6; residual H_2O at δ3.38 is shown off-scale; the peaks at ca. δ1.25 are associated with residual grease).

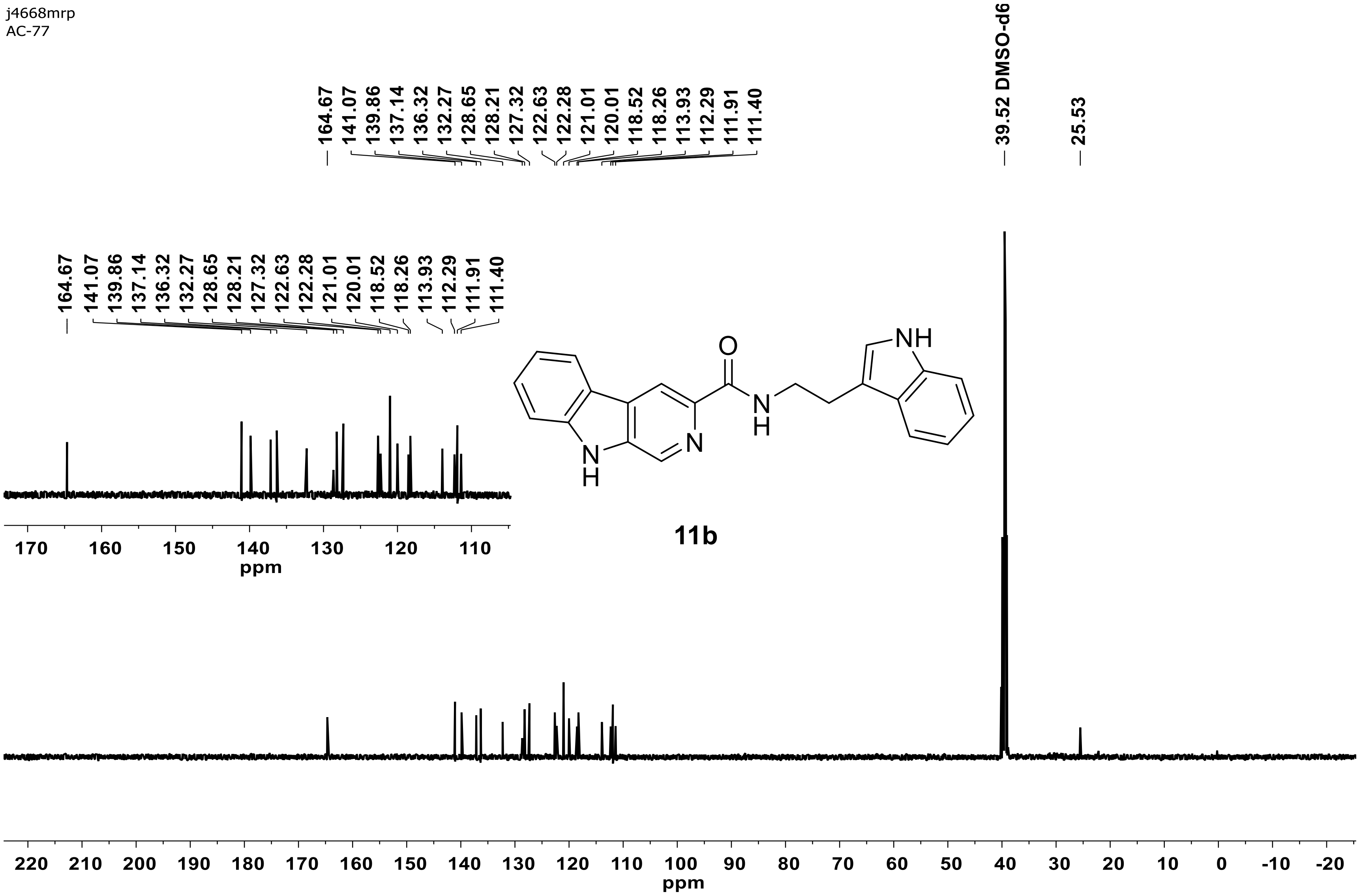

^{13}C NMR spectrum of compound 11b (DMSO-d_6).

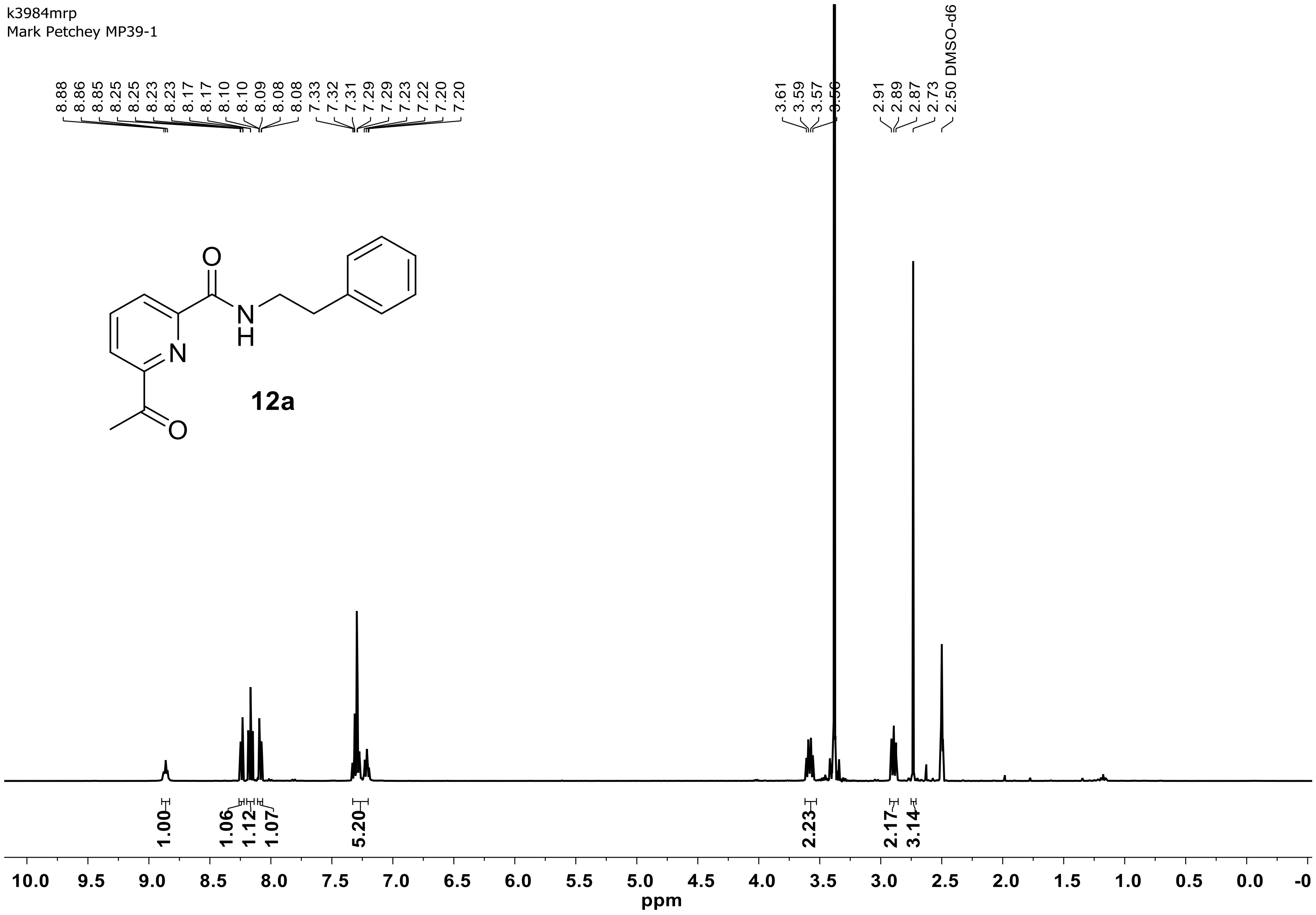

^{1}H NMR spectrum of compound 12a (DMSO-d_6).

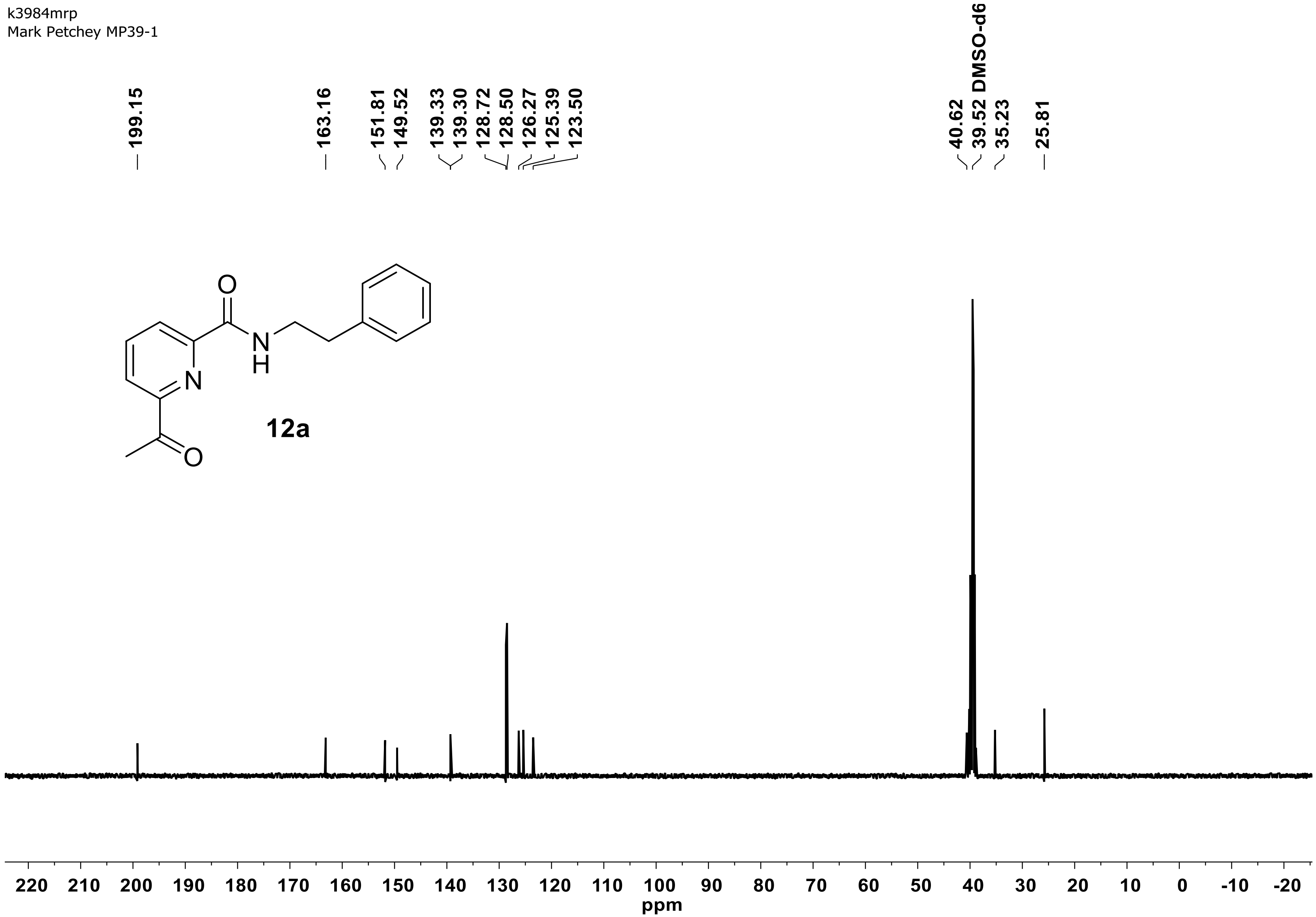

^{13}C NMR spectrum of compound 12a (DMSO-d_6).

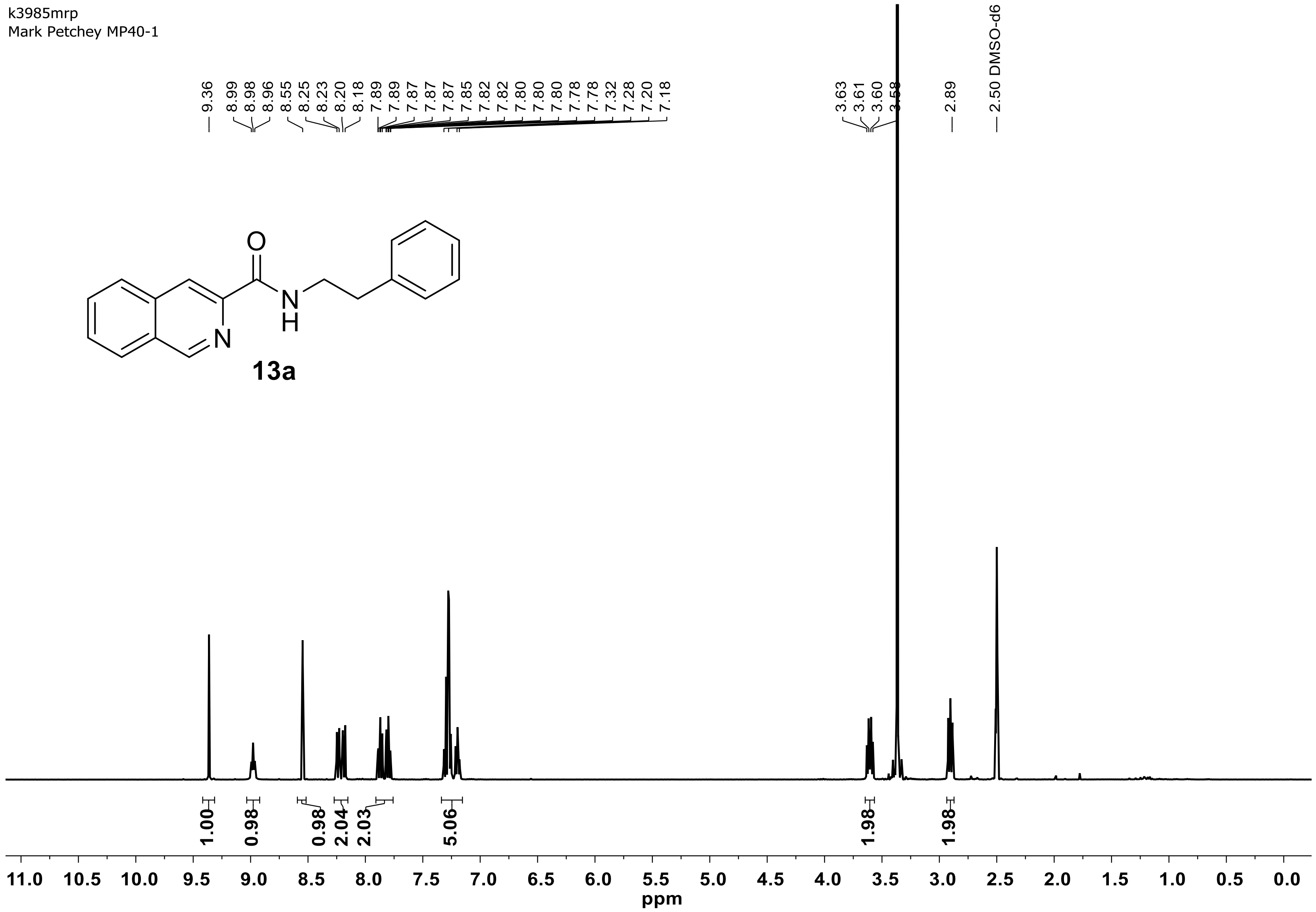

[1] H NMR spectrum of compound 13a (DMSO-d_6).

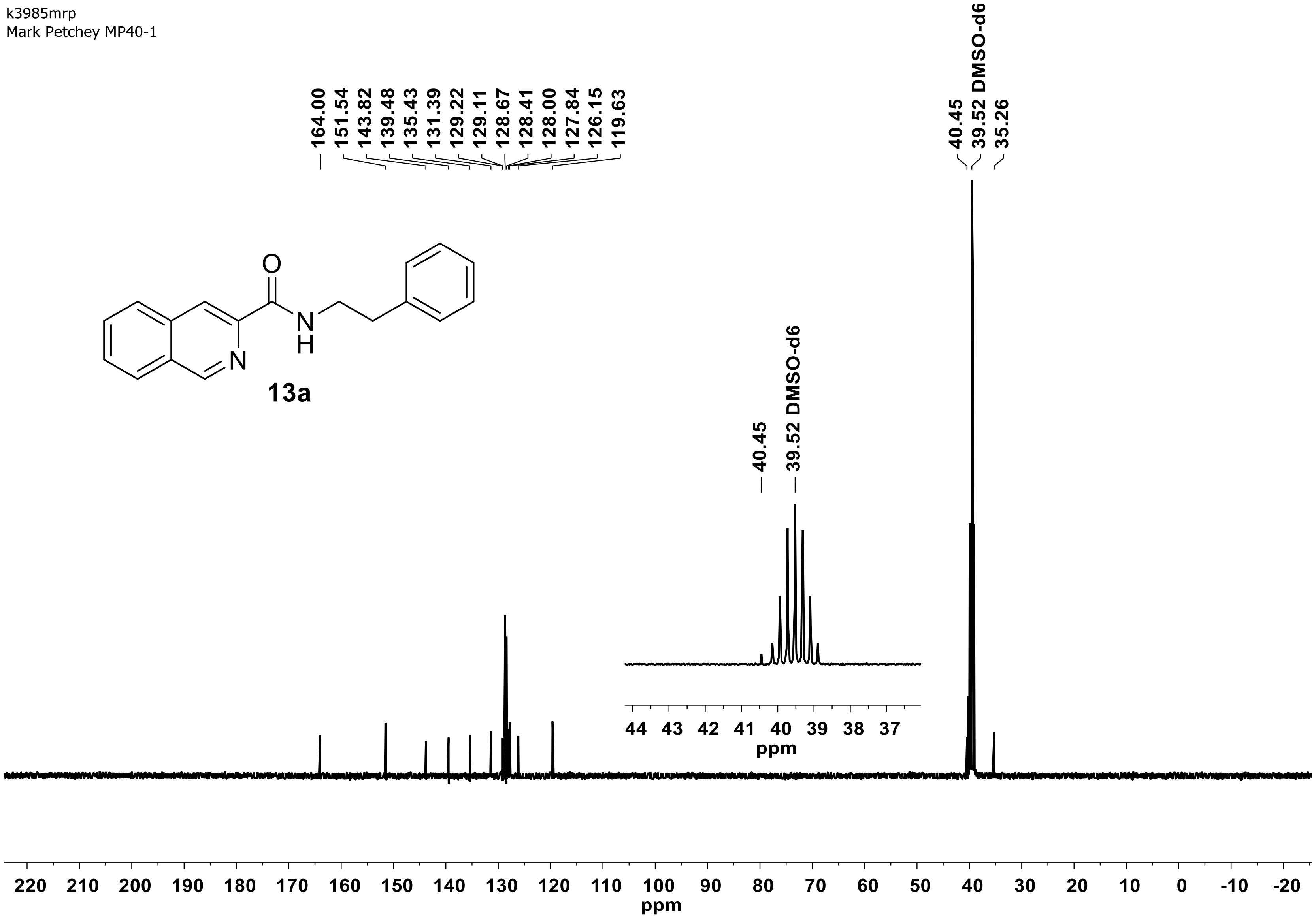

^{13}C NMR spectrum of compound 13a (DMSO-d_6).

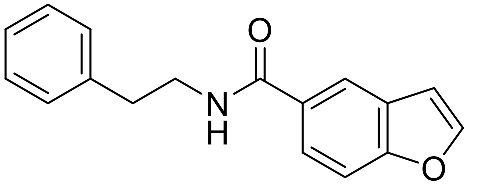

17a

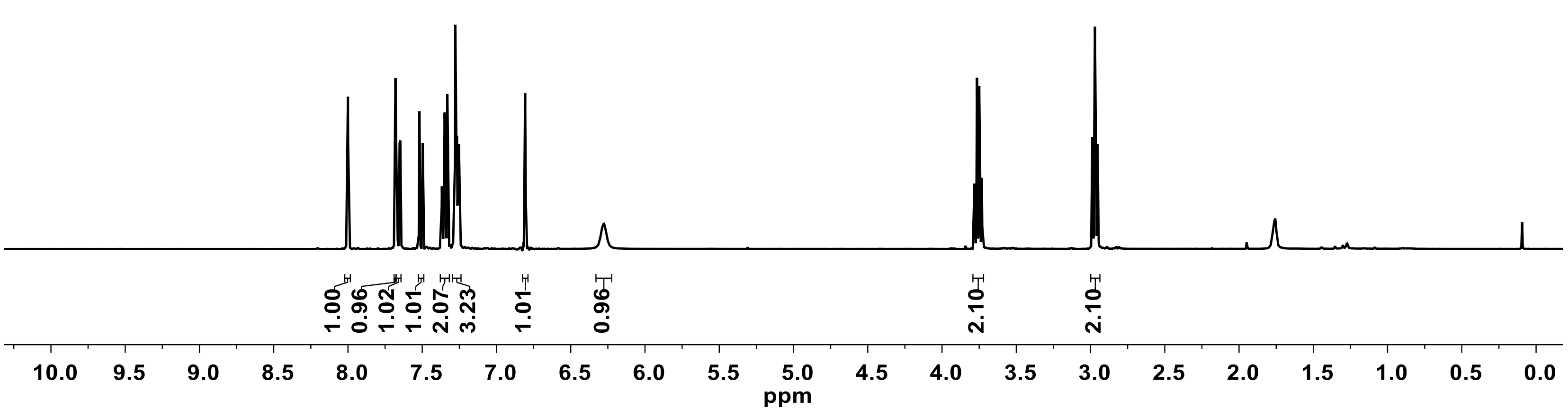

^{1}H NMR spectrum of compound 17a ($CDCl_3$; referenced to residual DCM residual CH_2Cl_2 at δ5.31).

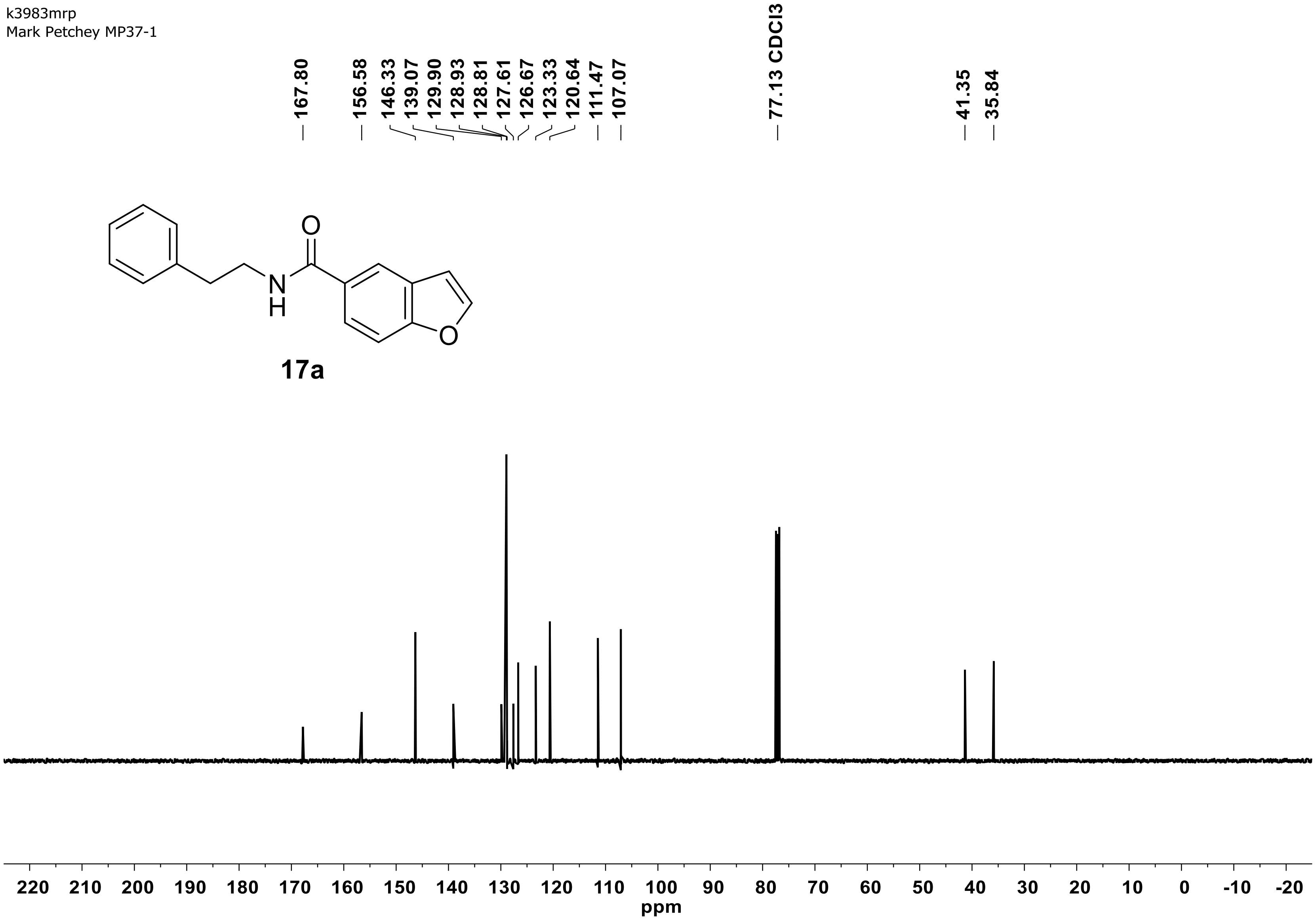

^{13}C NMR spectrum of compound 17a ($CDCl_3$).
